# Effects of sediment exposure on corals: a systematic review of experimental studies

**DOI:** 10.1186/s13750-022-00256-0

**Published:** 2022-02-07

**Authors:** Lillian J. Tuttle, Megan J. Donahue

**Affiliations:** 1 Hawai‘i Institute of Marine Biology, University of Hawai‘i at Mānoa, Kāne‘ohe, HI 96744 USA; 2 NOAA NMFS Pacific Islands Regional Office, Honolulu, HI 96860 USA

**Keywords:** Sedimentation, Turbidity, Marine management, Bleaching, Mortality, Sublethal physiology, Regulatory processes, Tipping points, LOAEL, NOAEL

## Abstract

**Background:**

Management actions that address local-scale stressors on coral reefs can rapidly improve water quality and reef ecosystem condition. In response to reef managers who need actionable thresholds for coastal runoff and dredging, we conducted a systematic review and meta-analysis of experimental studies that explore the effects of sediment on corals. We identified exposure levels that ‘adversely’ affect corals while accounting for sediment bearing (deposited vs. suspended), coral life-history stage, and species, thus providing empirically based estimates of stressor thresholds on vulnerable coral reefs.

**Methods:**

We searched online databases and grey literature to obtain a list of potential studies, assess their eligibility, and critically appraise them for validity and risk of bias. Data were extracted from eligible studies and grouped by sediment bearing and coral response to identify thresholds in terms of the lowest exposure levels that induced an adverse physiological and/or lethal effect. Meta-regression estimated the dose–response relationship between exposure level and the magnitude of a coral’s response, with random-effects structures to estimate the proportion of variance explained by factors such as study and coral species.

**Review findings:**

After critical appraisal of over 15,000 records, our systematic review of corals’ responses to sediment identified 86 studies to be included in meta-analyses (45 studies for deposited sediment and 42 studies for suspended sediment). The lowest sediment exposure levels that caused adverse effects in corals were well below the levels previously described as ‘normal’ on reefs: for deposited sediment, adverse effects occurred as low as 1 mg/cm^2^/day for larvae (limited settlement rates) and 4.9 mg/cm^2^/day for adults (tissue mortality); for suspended sediment, adverse effects occurred as low as 10 mg/L for juveniles (reduced growth rates) and 3.2 mg/L for adults (bleaching and tissue mortality). Corals take at least 10 times longer to experience tissue mortality from exposure to suspended sediment than to comparable concentrations of deposited sediment, though physiological changes manifest 10 times faster in response to suspended sediment than to deposited sediment. Threshold estimates derived from continuous response variables (magnitude of adverse effect) largely matched the lowest-observed adverse-effect levels from a summary of studies, or otherwise helped us to identify research gaps that should be addressed to better quantify the dose–response relationship between sediment exposure and coral health.

**Conclusions:**

We compiled a global dataset that spans three oceans, over 140 coral species, decades of research, and a range of field- and lab-based approaches. Our review and meta-analysis inform the no-observed and lowest-observed adverse-effect levels (NOAEL, LOAEL) that are used in management consultations by U.S. federal agencies. In the absence of more location- or species-specific data to inform decisions, our results provide the best available information to protect vulnerable reef-building corals from sediment stress. Based on gaps and limitations identified by our review, we make recommendations to improve future studies and recommend future synthesis to disentangle the potentially synergistic effects of multiple coral-reef stressors.

**Supplementary Information:**

The online version contains supplementary material available at 10.1186/s13750-022-00256-0.

## Background

Half of the world’s coral reefs have been lost in recent decades [1–4], while rising sea surface temperatures and local stressors threaten a third of those remaining [5]. This decline imperils the ecosystem services and economic value that reefs provide [6, 7]. Corals are protected around the world, and in the United States in particular, as federal trust resources, for their value as habitat for fish, and because some corals are listed as threatened or endangered species [8–10]. The regulatory programs that apply to coral reefs manage a wide variety of local stressors that include physical destruction or alteration, water quality, and point sources of thermal pollution [8–10]. Other regulatory programs are designed to conserve species that use coral reefs as habitat and indirectly benefit reefs [11].

Management of coastal activities can minimize the degradation of water quality and bottom habitat and, thus, mitigate reef decline in the face of climate change [12, 13]. However, reefs face a litany of local stressors that may act synergistically and thus complicate regulatory programs [14]. Among the most damaging pollutants on coral reefs is sediment, which can remain suspended in the water or be deposited on the coral surface and can contain toxicants, pathogens, and nutrients, all of which impact coral health [15–18]. There is enormous variation in the levels of exposure to deposited and suspended sediment that corals can tolerate, which may result from taxonomic differences, geographic location, sediment type, and exposure concentration, duration, and frequency. Exploring potential sources of this variation will help to quantify synergistic effects and identify critical threshold values for sediment and other anthropogenic stressors on reef-building corals, thus enhancing efforts to conserve and restore coral reefs.

**Fig. 1:**
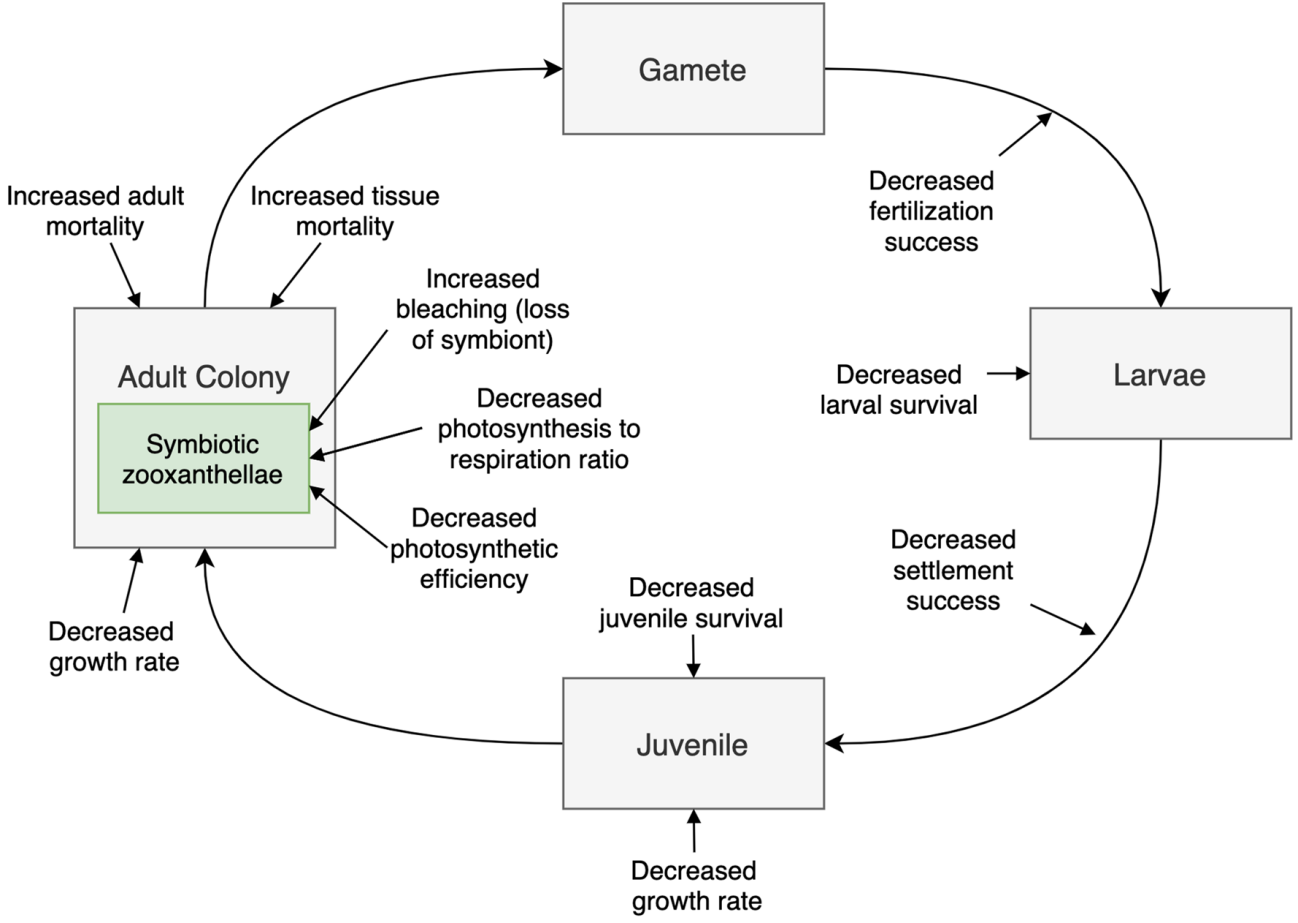
A simplified coral life history. Developmental stages in gray boxes and the coral endosymbiont in the green box. Hypothesized and previously documented biological responses to sediment are shown with arrows pointing toward the affected life-history stage/process

Sediment can affect corals throughout their life cycle (Fig. 1). High levels of sediment exposure may depress coral health, condition, and survival along multiple mechanistic pathways (reviewed in [15]). First, light attenuation reduces photosynthesis by symbiotic algae, thus limiting corals’ primary energy source. Also, corals divert available energy toward sediment clearance behaviors such as mucus production/sloughing and tentacle movement, which can interfere with filter feeding. Thus, sediment may lead to sublethal responses, such as reduced rates of growth, productivity, and calcification, as well as bleaching, disease susceptibility, physical damage (e.g., breaking and abrasion), and inability to regenerate following tissue damage [16, 19–22]. As the stress level intensifies, corals may experience lethal effects including tissue necrosis and colony death, which if widespread, may lead to changes in coral-reef community structure [23] and a decrease in ecosystem services.

Sediment exposure also affects the early life history of corals. Sediment may affect reproductive success by interfering with gamete fertilization in the water column [24]. Even relatively thin layers of sediment not harmful to most adult corals may inhibit coral larvae from settling on otherwise suitable surfaces [23, 25–27]; this can limit reef regeneration and persistence. Tolerance to sedimentation is estimated to be an order of magnitude lower for coral recruits than for adults [16], leading to high recruit mortality in areas of moderate to heavy sediment exposure.

Water quality guidelines for sediment often rely on the results of previously published literature syntheses concerning the effects of coastal development and terrestrial runoff on coral reefs, the most widely cited of which are more than a decade old [16, 18]. Substantial new experimental data are now available to inform best management practices. More recent syntheses of the effects of sediment on corals [15, 17, 28, 29] provide qualitative accounts only, thus providing a starting point for the quantitative synthesis that allows regulatory assessments to rigorously identify thresholds and quantify adverse effects.

In response to needs identified by the Pacific Islands Regional Office (PIRO) of the U.S. Department of Commerce’s National Oceanic and Atmospheric Administration (NOAA), we conducted a systematic review and meta-analysis that identified thresholds of coral response to both deposited and suspended sediment, bringing to bear the most current and comprehensive information for regulatory decision-making. Specific research questions and a protocol were developed by an advisory team at NOAA in conjunction with a research team from the University of Hawai‘i [30]. The UH research team then conducted the systematic review and meta-analysis, the results of which we report here, with minimal changes to the original protocol (see *Methods*).

## Objective of the review

The primary objective of the present study is to perform a systematic review of peer-reviewed, publicly available, and grey literature to develop thresholds for suspended and deposited sediment stressors that affect nearshore coral-reef ecosystems. We followed established methodologies [31–33] for systematic review in environmental management to (a) identify, collect, and evaluate sources of empirical data on the effects of sediment on corals; (b) extract relevant data from these sources; and (c) use statistical and meta-analytic procedures to identify stressor thresholds on coral reefs.

The population of interest was reef-building corals (order Scleractinia) exposed to either suspended and/or deposited sediment in comparison to experimental controls. Outcomes of interest were physical, physiological, behavioral, developmental, and ecological responses of corals associated with sediment exposure. To avoid the confounding effects of uncontrolled stressors that typically co-occur in observational studies, we focused on experimental studies that quantify the causal relationship between sediment and coral response. Monitoring and other observational studies were used to contextualize experimental findings. We address the following overarching question and sub-questions:

How does sediment exposure affect corals?

1. *Types of effects*: What physical, physiological, behavioral, developmental, and ecological responses of corals (e.g., mortality, tissue necrosis, growth rate, photosynthetic yield) are associated with sediment exposure?
2. *Effect sizes and thresholds*: What is the relationship between sediment exposure (concentration, duration, or frequency of suspended or deposited sediment) and coral response?
  - What is the effect size (magnitude or frequency) of coral response as a function of sediment exposure?
  - What is the threshold of sediment exposure above which coral responses are detected?
3. *Effect modifiers*: How do coral responses to sediment exposure differ by geography, sediment type, and coral taxonomy, morphology, and developmental stage?

## Methods

Our systematic review and meta-analysis was conducted according to the Guidelines and Standards for Evidence Synthesis in Environmental Management, version 5.0 [31, 32] and reported according to the procedures of ROSES (RepOrting standards for Systematic Evidence Syntheses) [33, Additional file 1].

### Deviations from the protocol

Few deviations were made from the previously published, a priori systematic review protocol [30]. Given that we employed a small team, we provide more detail about how we ensured repeatable and consistent decisions during different stages of the review, especially article screening, data extraction, and study validity assessment. We reframed our study objectives into three major categories (types of effects, effect sizes and thresholds, and effect modifiers; see above), and thus reframed our Data Synthesis and Presentation to reflect these objectives. Our narrative synthesis illustrates how sediment exposure (concentration and duration), coral taxonomy, and coral life-history stage shape biological responses to sediment stress (Objective 1). At the request of our advisory team, we include lowest-observed and no-observed adverse-effect levels (LOAELs, NOAELs), which are common thresholds used in regulatory environments, and which serve as estimates of the exposure levels at which we expect sediment to begin adversely affecting corals (Objective 2b). We also provide substantially more details concerning our meta-analytical methods, which use effect sizes to quantify the relationship between sediment exposure levels and the magnitude of corals’ response (Objectives 2a, 2b, and 3).

### Search for articles

Our systematic review started with the definitive reviews on the subject, which include Rogers [18], Fabricius [16], Erftemeijer et al*.* [15], Risk [29], and Jones et al*.* [17, 28]. We developed a list of potential sources of data, hereafter called ‘benchmark studies,’ from this set of reviews [Additional file 2].

#### Search sources

To supplement this list, we conducted electronic literature searches using the following databases or search engines (DSE) using the University of Hawaiʻi at Mānoa Library: (1) *ISI Web of Science* (*All Databases*, see Table 1), (2) *JSTOR*, (3) *Aquatic Sciences and Fisheries Abstracts*, (4) *Dissertations and Theses Global*, (5) *James Cook University Library One Search*, (6) *ReefBase’s Proceedings of the International Coral Reef Symposium*, (7) *Science.gov*, (8) *Great Barrier Reef Marine Park Authority (GBRMPA) Elibrary*, and (9) *Western Australia Marine Science Institute’s Dredging Science Node (WAMSI DSN) repository*. These DSE are categorized and described in Table 1, along with search specifications (e.g., full text vs. abstract only, date ranges, dates of searches) for each. DSE 1–3 target peer-reviewed literature produced by commercial publishers, while DSE 3–9 target ‘grey’ literature, including theses/dissertations, conference proceedings, and reports for governmental/non-governmental entities.

**Table 1:** Search specifications for each database or search engine (DSE)

DSE category	DSE Name (Abbrev.)	DSE scope	Search specification(s)	Search date
Bibliographic databases:	1) *Web of Science* (WoS)*, All Databases*	General science	Topic (titles, authors, abstracts, keywords); ‘All Databases’ include: (a) WoS Core Collection (SCI-EXPANDED, ESCI), (b) Biological Abstracts, (c) SciELO Citation Index, & (d) Zoological Record	All years (1950–May 23, 2019)
	2) *JSTOR*	General academic	Abstract, All content, Any time	May 23, 2019
	3) *Aquatic Sciences and Fisheries Abstracts* (ASFA)	Aquatic and marine science	Abstract, Any time	May 23, 2019
	4) *Dissertations & Theses Global* (PQDT)	Global dissertations and theses	Abstract, Any time	May 23, 2019
Organizational databases:	5) *James Cook University One Search* (JCU)	Australian university dissertations and theses	Abstract, Dissertation/Thesis, Any time	May 23, 2019
	6) *ReefBase*	Proceedings of the International Coral Reef Symposium	Title; also Keywords for taxon-specific search terms; Any time	Nov. 4, 2019*
	7) *Science.gov*	United States federal government science	Full record (no 'Abstract' option), Any time	May 23, 2019
	8) *Great Barrier Reef Marine Park Authority (GBRMPA) Elibrary*	Australian federal government science	All of ELibrary, Type = Report, Any time	May 23, 2019
	9) *Western Australia Marine Science Institute’s Dredging Science Node (WAMSI DSN)*	Australian non-governmental reports	All reports and research articles listed at [34], Any time	Dec. 16, 2019**

*Search not sensitive to search date between publications of conference proceedings every 4 years (2017–2022)

**Search not sensitive to search date because no new reports and research articles were posted to this site between May, 23 2019 and Dec. 16, 2019

#### Search terms and strings

In developing the structure of this systematic review, we adopted the ‘PECO’ approach [35], which defines the relevant Population (including species), Exposure, Comparator, and Outcomes as pillars of the research question and serve as inclusion/exclusion criteria during the screening process. For ‘Population,’ the following genera were specifically important because they contain species that are identified by the U.S. Endangered Species Act as either threatened or endangered [10]: *Acropora, Anacropora, Cantharellus, Dendrogyra, Euphyllia, Isopora, Montastraea, Montipora, Mycetophyllia, Orbicella, Pavona, Porites, Seriatopora, Siderastrea,* and *Tubastraea*. These additional genera were important because of their importance in the Pacific region under U.S. jurisdiction, as indicated by our NOAA advisory team: *Alveopora, Astreopora, Favia, Favites, Goniastrea, Goniopora, Leptastrea, Leptoria, Lobophyllia, Millepora, Platygyra, Pocillopora,* and *Turbinaria*. The following search string in English uses Boolean operators and wildcards to improve the quality (i.e., true positive results) of search results and was tested for its comprehensiveness [30], shown in Additional file 2 along with slight modifications to the syntax of the search term, which were necessary for different databases:

((coral AND sediment*) OR (coral AND suspend*) OR (coral AND turbidity) OR (coral AND mud) OR (coral AND terrigenous) OR (coral AND silt*) OR (coral AND plume) OR (coral AND dredg*) OR (coral AND land-based) OR (sediment* AND Acropora) OR (sediment* AND Anacropora) OR (sediment* AND Cantharellus) OR (sediment* AND Dendrogyra) OR (sediment* AND Euphyllia) OR (sediment* AND Isopora) OR (sediment* AND Montastraea) OR (sediment* AND Montipora) OR (sediment* AND Mycetophyllia) OR (sediment* AND Orbicella) OR (sediment* AND Pavona) OR (sediment* AND Porites) OR (sediment* AND Seriatopora) OR (sediment* AND Siderastrea) OR (sediment* AND Tubastraea) OR (sediment* AND Alveopora) OR (sediment* AND Astreopora) OR (sediment* AND Favia) OR (sediment* AND Favites) OR (sediment* AND Goniastrea) OR (sediment* AND Goniopora) OR (sediment* AND Leptastrea) OR (sediment* AND Leptoria) OR (sediment* AND Lobophyllia) OR (sediment* AND Millepora) OR (sediment* AND Platygyra) OR (sediment* AND Pocillopora) OR (sediment* AND Turbinaria)).

#### Search results, estimating the comprehensiveness of the search, and search limitations

Search results were saved as BibTeX (.bib) or RIS (.ris) files and imported into an open-source reference manager (*Mendeley*) with tools to identify and remove duplicates. We tested the thoroughness of our DSE searches by comparing the DSE search results with those of two other lists of potential sources of data. First, we queried *Google Scholar* with a truncated search string because *Google Scholar* would not accept our full search term due to its length. This truncated search string included Boolean search terms with “coral” but excluded those that were genus-specific [see Additional file 2 for search specifications]. We evaluated the titles and abstracts of the top 200 *Google Scholar* search results to include only potentially ‘eligible’ articles (see *Screening Process*, below), and removed articles that were duplicated in the DSE search. We were then left with potentially eligible studies that the DSE search had not found. Similarly, we screened the list of benchmark studies (described above) to include only potentially eligible, un-duplicated articles [Additional file 2]. We examined all potentially eligible, un-duplicated articles within the *Google Scholar* search and the list of benchmark studies to understand why they were not also found in the DSE search.

Based on any systematic patterns of bias that we discerned, we made our DSE more inclusive. For instance, to avoid regional/language biases, we included the SciELO Citation Index in the Web of Science search [DSE 1] that targets research in Latin America, including many Caribbean countries where we expected relevant work to be based. Of the 200 *Google Scholar* search results, 16 (8%) were un-duplicated in the DSE search and deemed potentially eligible based on preliminary title and abstract review. However, two could not be located and the remaining 14 were reclassified as ‘ineligible’ upon review of the full texts using PECO criteria (see *Eligibility criteria* for methods and Additional file 3 for justification). Of the 129 benchmark studies, 123 (95.3%) were found in the DSE search. Of the six benchmark studies that were not found in the DSE search [Additional file 2], one could not be located and five were reclassified as ‘ineligible’ upon review of the full texts using PECO criteria [Additional file 3]. There were no obvious, systematic reasons for these sources’ exclusion from the DSE search and none passed the criteria necessary to be included in our review and meta-analysis, so we considered our search to be relatively comprehensive.

### Article screening and study eligibility criteria

#### Screening process

For the purposes of this systematic review, an “article” is defined as any written document including scientific papers, abstracts, reports, book chapters, theses/dissertations, and other publications. Unique articles were imported into *abstrackr* [36], a free web application in which the results of a literature search for a systematic review are uploaded, organized, and screened. All reviewers independently screened a pilot round of 100 articles (titles and abstracts evaluated together), classified each as ‘eligible,’ ‘ineligible,’ or ‘maybe eligible’ to address the research question. The reviewers discussed any discrepancies in their decisions and further clarified, revised, and agreed upon the classification criteria until a consensus was reached for each conflict. Subsequently, all articles (n = 10,221) were independently screened by at least two reviewers, and any conflicts between the two reviewers were resolved by a third member of the review team. If a potential article was authored or co-authored by a reviewer, then two other reviewers determined the potential eligibility of the article. This was done during the assessment of study validity and the full-text screening as well (see below). Post-hoc consistency checks revealed that after the pilot round, fewer than 4% of articles had conflicting decisions. Approximately 1.2% (n = 120) were discrepancies between an article being eligible and ineligible, 1.3% (n = 134) were between ineligible and maybe eligible, and 1.4% (n = 147) were between eligible and maybe eligible.

As reviewers make repeated decisions about article relevance, *abstrackr*’s machine learning protocol predicts eligible articles and presents them to the reviewer(s) in order from ‘most likely’ to ‘least likely’ to be eligible. This can increase workload savings while maintaining relatively high sensitivity and specificity and relatively low false-negative rates, thus making it a useful addition to the screening process [37, 38]. Regardless of *abstrackr*’s prediction, the reviewer(s) screened all titles and abstracts. We considered English abstracts for non-English full texts during the article screening process. When a non-English article was deemed potentially eligible, we searched for translations of full texts. If English translations were not available, the article was not screened but noted as non-English in Additional file 3.

The full texts for all ‘eligible’ and ‘maybe eligible’ articles were collected and reviewed according to the ‘Eligibility Criteria’ described below. Two reviewers individually screened 10% of the full texts (n = 40 of 396) and compared their decisions. At this stage, there were no conflicting decisions between the two reviewers and so the second reviewer did not need to screen an additional 10% of full texts (as originally proposed in the protocol [30]). Thereafter, full texts were screened by individual reviewers.

Each article may report the results of multiple studies. We defined a “study” as a manipulative experiment that addresses a single hypothesis or research question. In the case of articles containing multiple studies, each study was independently reviewed according to the ‘Eligibility Criteria.’

In the particular case of dissertations and theses, special care was taken to ensure that there was no duplication in our review between dissertation/thesis chapters and publications based on the same data. Peer-reviewed publications and final reports took precedence over dissertation/thesis chapters of the same data. When dissertation/thesis chapters provide additional data that were not reported in the peer-reviewed document, these data supplemented that of the peer-reviewed document but remained a part of the same ‘study.’ Relevant, unpublished chapters were treated as independent studies. There were no cases in which a study spanned multiple articles.

#### Eligibility criteria

The PECO framework is useful in defining which populations, exposures, comparisons, and outcomes should be included or not in a systematic review and meta-analysis [35]. We used this framework to determine the inclusion or exclusion of each article for further review and analysis at the stages of title/abstract and full-text screening, though some criteria were not evident until the full-text review. To be included, an article had to meet every criterion. Otherwise it was excluded.

*Population* All life stages of all shallow (photic zone, ≤ 80 m depth) scleractinian coral genera in all warm-water ocean basins (20–30 °C).

*Exposure* Exposure to concentrations of suspended and/or deposited sediment of marine or terrigenous origin. For manipulative experiments conducted in either the field or laboratory, this was the application of suspended or deposited sediment.

*Comparison* Specimens experimentally exposed to suspended or deposited sediment must be compared to an appropriate experimental control in either the field or laboratory.

*Outcome(s)* Specific endpoints are all physical, physiological, behavioral, developmental, and ecological responses of corals associated with exposure to deposited and/or suspended sediment. These may include but are not limited to tissue or colony mortality, bleaching, and changes in rates of growth, photosynthesis, and larval settlement or survival. Outcomes were recorded as binary or continuous data, as reported in the study.

*Eligible types of study design* Quantitative meta-analysis were limited to the results of experimental studies that quantify the cause-effect relationship between sediment stress and coral response (including BACI-designed studies, and those conducted in the field or laboratory, mesocosms, etc.), compared to the response of corals to ‘ambient’ or ‘control’ conditions. Observational studies were identified and when informative as a means to contextualize the findings of manipulative experiments, were included in the narrative synthesis.

### Study validity assessment

We critically appraised all studies that passed the full-text screening process using a number of parameters including the following, which may affect a study’s external validity:

- Study setting: field or laboratory;
- Temporal extent of the study: relatively long-term monitoring or short-term measurements;

and the following, which may affect both the external and internal validity of a study:

- Study design: manipulative or observational study; presence/extent of pseudoreplication;
- Randomization: how sediment exposure levels were assigned to coral samples; and
- Confounding factors: degree of accounting for potential effect modifiers, if present.

Internal validity was further assessed per the criteria outlined by Bilotta et al*.* [39], which adapted Cochrane’s ‘risk of bias’ tool [40] for environmental science applications. This “Environmental-Risk of Bias Tool” assesses selection, performance, attrition, reporting, and miscellaneous biases. With this information, we also used the “Environmental GRADE Tool” [39] to determine the overall quality (high, moderate, low, or very low) of each study. We found no studies with a low or very low overall GRADE (indicative of high susceptibility to bias) requiring exclusion from further analysis. Prior to full-text review and for a pilot round of five studies, quality was assessed by the entire review team. The specific questions and criteria we used to assess risk can be found in Additional file 3. Conflicting decisions of a study’s quality (differing GRADE) were resolved by the entire review team by discussing a study’s putative sources of bias. To ensure repeatability of quality assessment after the pilot round, the review team iteratively assessed an additional five studies until no conflicts emerged. There were no conflicting decisions after the second round of five studies, so the validity and quality of each remaining study was assessed by no more than one reviewer.

We used these critical appraisals and tools to organize studies into groups of comparable records across which we should (and should not) meta-analyze. This process determined the scope of inference of our meta-analysis, thus defining the extent to which our results applied to the diverse set of sedimentation events that occur on coral reefs.

### Data coding and extraction strategy

Information from studies was input into a data coding and extraction form [30] and recorded in a project database [Additional file 3]. The database includes meta-data (e.g., author(s), year published, location published, etc.) and study characteristics such as the sample sizes, means, and variations of coral response(s) to sediment and control conditions. When these data were not reported in the text, we extracted them from figures using open-source digitizing software that convert graph images into numerical data (Datathief III [41]). When only raw data were available, we calculated summary statistics (sample mean, standard deviation, standard error). When information was indecipherable or missing, we contacted the corresponding author of the study for clarification. In a pilot round, all reviewers extracted data from the same three studies, compared their results for any inconsistencies, and adjusted the protocol to improve the consistency of the data extraction process. After this pilot round, each study had data extracted independently by one reviewer. Thereafter, progress and questions were discussed by the entire review team on a weekly basis until data extraction was complete, which promoted consistency among team members. There were no major inconsistencies in data extraction after the pilot round, so regular meetings served to identify and correct minor inconsistencies (e.g., number formatting, unit conversion and notation, capitalization, spelling, etc.) as data extraction progressed.

### Potential effect modifiers/reasons for heterogeneity

There are several factors that may cause variation in measured outcomes, information about which was extracted and recorded in the project database [Additional file 3]. The list of effect modifiers includes the following: study location (ocean basin, region, and site), study species and morphological form (e.g., massive, plating, branching), time/season of sediment-exposure event, sediment composition (e.g., silt–clay vs. calcareous sand) and provenance (terrestrial vs. marine), sediment dose/concentration (and methods for measuring dose), sediment exposure duration, and possible interacting effects (e.g., light attenuation in concurrence with suspended sediment, or nutrient-enriched deposited sediment). Effect modifiers were either categorical or numerical, and some required conversion of reported units to a common standard (e.g., for deposited sediment the standard is mg/cm^2^/day and for suspended sediment it is mg/L). While some effect modifiers were explicitly addressed in our meta-analysis (study species and sediment exposure concentration/duration; see “8”), others suffered from inconsistent reporting and/or insufficient replication (see “13”). To the extent possible, we report narrative information from these other sources of heterogeneity in *Review findings*.

### Data synthesis and presentation

Our narrative synthesis includes the results of all eligible studies and summarizes the scope of existing studies by population and exposure to (1) deposited sediment or (2) suspended sediment, and by study design. To address Review Objective 1 (Types of Effects), we describe the range of biological mechanisms of coral response to sediment exposure across life-history stages and construct tables that illustrate the responses of corals organized by sediment exposure (concentration and duration), coral taxonomy, and coral life-history stage.

#### LOAELs and NOAELs

**Fig. 2:**
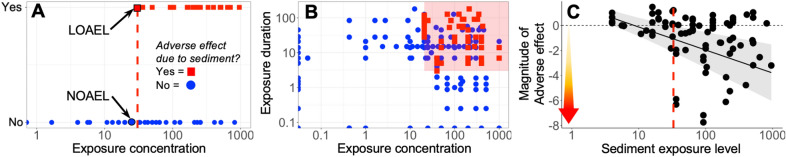
Graphical summary of threshold synthesis and meta-analysis endpoints: detection of adverse effects that identifies exposure thresholds to (**A**) sediment concentration and (**B**) sediment concentration and duration, jointly; and analysis of continuous data that (**C**) characterizes the relationship between exposure and the magnitude of an adverse effect (where zero is no adverse effect with respect to control). The red, dashed line in **A** represents the LOAEL and in **C** represents the dose–response threshold, where the confidence interval (in gray) no longer overlaps with zero. The red, rectangular area in **B** is bounded by the LOAELs for concentration and duration, thereby representing the exposure conditions under which adverse effects have been observed in studies from our review

To address Review Objective 2b (Effect Thresholds), our advisory team requested that we identify thresholds of coral response to both deposited and suspended sediment, using threshold types that are commonly used in toxicological and other regulatory contexts (Fig. 2A, B):

1. *LOAEL*—the ‘lowest-observed adverse-effect level,’ i.e., the lowest exposure level at which an adverse effect was detected, and
2. *NOAEL*—the ‘no-observed adverse-effect level,’ i.e., the highest exposure level at which an adverse effect was NOT detected.

For the purposes of the coral-specific analyses presented herein, we define *adverse effect* as any response of a coral individual, colony, or treatment group that may negatively affect the coral’s fitness and/or survival. These adverse effects may include physiological changes (e.g., reduced growth or photosynthetic rates), bleaching, tissue necrosis, and colony mortality. This definition is independent of response magnitude; while the effect may potentially reduce a coral’s fitness, the reduction in fitness may not be measurable.

To identify LOAELs and NOAELs of sediment exposure, we first classified each study by the presence or absence of a detected effect. We thus compared corals exposed to sediment (treatment group) with corals not exposed to sediment (control group) from the same study. If the original study detected a statistically significant decline in condition of the treatment group as compared to the control group, then that treatment group was coded as a ‘1’ (presence of adverse effect). Conversely, if the treatment group was not significantly different from the control group (or fared better), then it was coded as a ‘0’ (absence of adverse effect). When articles had more than one treatment group (e.g., for multiple sediment concentrations), each treatment group was compared and coded as described above. This information was summarized (Fig. 2A, B) to visualize the range of exposure concentrations and durations assessed for each adverse effect and to identify the LOAELs and NOAELs. Note that this is a graphical summary of existing studies and does not control for differences in power between studies (which are controlled for in the Dose–Response Meta-Analysis).

#### Dose–response meta-analyses

Studies reported many different types of coral responses to deposited and suspended sediment stress, the majority of which were reported as continuous variables (e.g., photosynthetic efficiency, growth rate, larval settlement rate, etc.). To address Objective 2a (Effect Sizes), we calculated the standardized difference in means for each treatment group within each study. We calculated this effect size using Hedges’ *d* and the variance, *s*, thereof [42] which is unaffected by unequal sampling variances in the paired groups (e.g., treatment and control conditions) and includes a correction factor (*J*) for small sample sizes:

$$d=\frac{({\overline{X} }_{T}-{\overline{X} }_{C})}{s}J, \quad J=1-\frac{3}{4\left({n}_{T}+{n}_{C}\right)-9}, \quad s=\sqrt{\frac{\left({n}_{T}-1\right){SD}_{T}^{2}+\left({n}_{C}-1\right){SD}_{C}^{2}}{{n}_{T}+{n}_{C}-2}}$$

where $\overline{X }$ is the sample mean, *T* and *C* are treatment and control groups, respectively, *SD* is standard deviation, and *n* is sample size. We made funnel plots of the effect size (*d*) plotted against sample size (*n*_*T*_) to detect possible publication bias [43, 44]. Relatively asymmetric funnel plots indicate greater risk of publication bias. We thus include in our results interpretations of these diagnostic plots for each of the statistically significant meta-analyses (described below), including descriptions of outliers that may provide insight into potential effect modifiers. Similarly, we report I^2^ as an estimate of residual heterogeneity for each best-fit model, i.e., the percentage of variance in a meta-analysis that is attributable to heterogeneity among dose–response comparisons within a study. Heterogeneity is considered substantial when I^2^ is above 75%.

We then explored the relationship between effect size (*d*) and stressor intensity with hierarchical mixed-effects models that fit exposure–response curves (‘dose–response meta-analysis,’ or DRMA, models, Fig. 2C). This model structure allowed us to examine the overall effects on corals while accounting for within- and between-study (co)variance structures (e.g., due to random effects and other effect modifiers). We fit unique models for each of the coral responses converted to effect size. These responses were always specific to a coral age class. For instance, fertilization success rate is specific to coral gametes and settlement rate is specific to larvae. Therefore, we did not combine analyses across coral age-classes.

All DRMA models were fit in *R* [45] with the *mixmeta* package and function [46]. Weights assigned to studies depended on the relative size of the within- and between-study covariance matrices reported as components in *mixmeta* objects. We adopted a ‘fixed slope, random intercept’ approach and fit models with three possible fixed-effects structures: (1) exposure concentration (reduced model), (2) concentration and duration (reduced model), and (3) concentration, duration, and the interaction between the two (full model), and five possible random-effects structures: (1) species, (2) study, (3) study nested within article, (4) species and study, and (5) species and study nested within article. Model selection followed Zuur et al. [47], whereby we compared model structures using Akaike Information Criterion (AIC) and likelihood ratio tests. To determine the best random-effects structure (of the five listed above), we compared full fixed-effects models with different random effects using restricted maximum likelihood (REML). Once a random-effects structure was chosen, we determined the best fixed-effects structure (of the three listed above) by comparing full vs. reduced models using maximum likelihood (ML). We inspected the residuals of best-fit models and, in all cases, used a log_10_ transformation of exposure concentration to conform with statistical assumptions and allow model convergence.

We chose this combination of fixed effects because sediment exposure levels were the key stressors of interest in determining thresholds, and because other effect-modifiers—including study location, season, sediment composition and provenance (see “7”)—had inconsistent reporting and/or insufficient replication to include as model variables (see *Review findings*). For similar reasons, species, study, and article were the only random effects considered despite other possible sources of heterogeneity.

To address Objective 2b (Effect Thresholds), we estimated the ‘dose–response threshold’ for a coral response as the exposure level at which the upper bound of the 95% confidence interval of a DRMA regression did not overlap with zero (red, dashed line in Fig. 2C). Since a value of zero indicates no difference between treatment and control groups, this threshold identifies the minimum exposure that produced a statistically significant difference between treatment and control groups.

## Review findings

### Review descriptive statistics

**Fig. 3:**
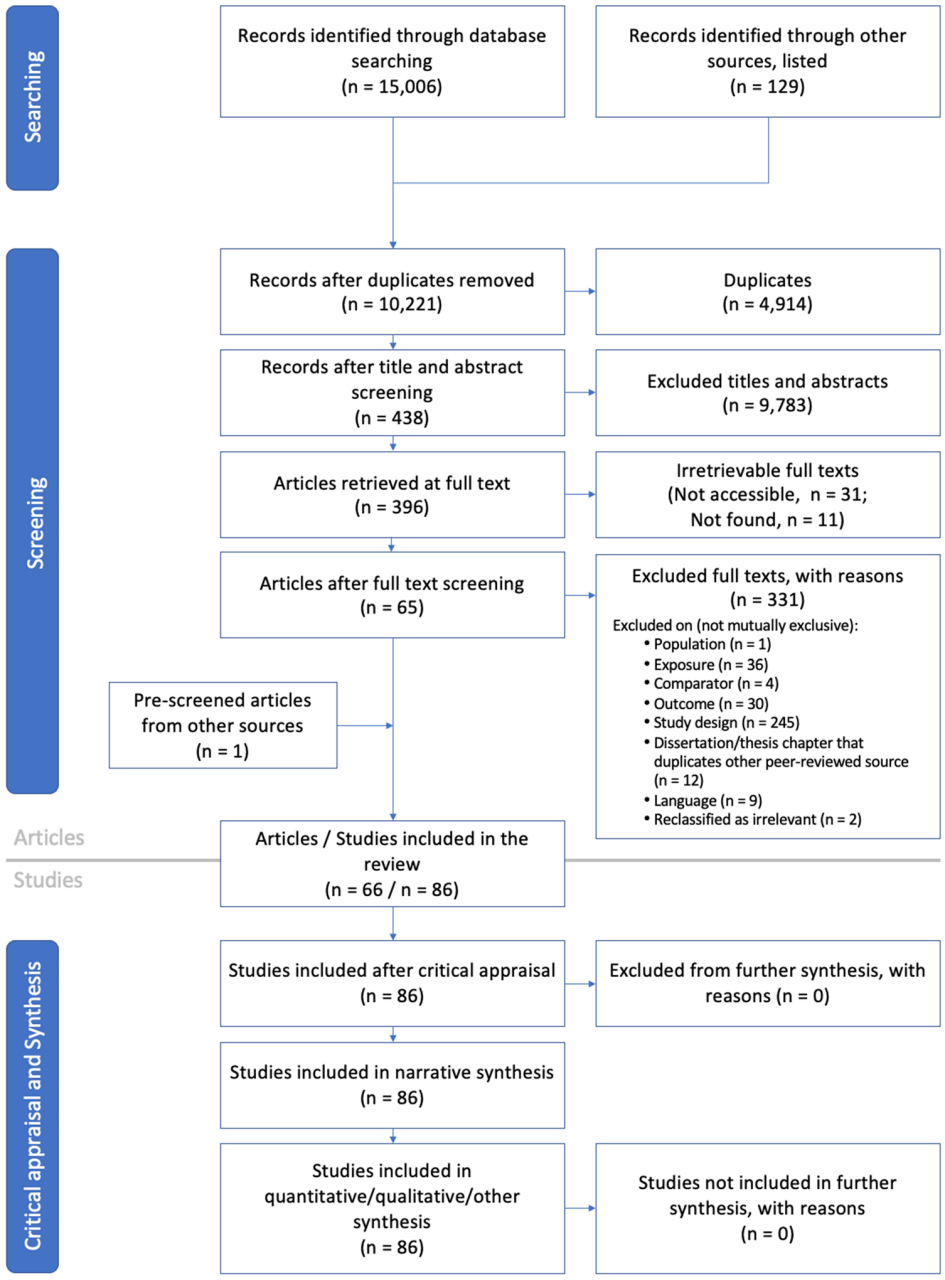
ROSES flow diagram for systematic reviews, modified Version 1. [33] https://doi.org/10.6084/m9.figshare.5897389; https://www.roses-reporting.com/systematic-review-reports. Accessed 11 August 2020. During the full-text review process we reclassified two articles as being ‘ineligible’ (from ‘maybe eligible’), indicating that they were false positives during the title/abstract screening process

In addition to 129 benchmark studies [Additional file 2] identified within the definitive reviews [15–18, 28, 29], our DSE searches returned 15,006 records (Fig. 3). After removing duplicates from these records, we screened the titles and abstracts of 10,221 records, 396 of which underwent a full-text screening, the results of which are reported in Additional file 2. Included in our review (narrative and data syntheses) are 65 articles, in which are the results of 86 studies (Fig. 3). Of these, we distinguish between studies that investigated the effects of deposited sediment, suspended sediment, and both deposited and suspended sediment on various responses of corals (Table 2). Because there was only one included article/study that quantified the effects of deposited and suspended sediment together [48], we do not conduct a meta-analysis of the synergistic effects of deposited and suspended sediment. Instead, we include this article/study in each of the separate analyses for deposited and suspended sediment.

**Table 2:** The number of articles and studies included in the review (narrative and data syntheses), by sediment category

	Deposited sediment	Suspended sediment	Deposited and Suspended Sediment	**Total**
Number of articles	44	22	1	65
Number of studies	45	42	1	86

The majority of articles in our review (73.8%, 48 of 65) were conducted in the Pacific Ocean, followed by 18.5% (12 of 65) in the Atlantic Ocean, 3.1% (2 of 65) in the Indian Ocean, and 4.6% (3 of 65) in the trans-oceanic boundary between the Indian and Pacific Oceans (i.e., Malacca Strait). This geographic bias toward the Pacific was expected because most of the world’s coral reefs and species are located in this region. The included articles were published between 1979 and 2018, with increasing frequency through the decades. The largest group of articles were published in the past decade since 2010 (40.0%, 26 of 65), followed by 29.2% (19 of 65) during the 2000s, 16.9% (11 of 65) during the 1990s, 12.3% (8 of 65) during the 1980s, and 1.5% (1 of 65) during the 1970s. For a full reference list of all articles included in our review (Tables 3, 4) and all those that were excluded at the full-text screening (with reasons for exclusion), see the project database [Additional file 3].

**Table 3:** All articles and studies included in the meta-analysis of the effects of deposited sediment (DSC) on corals

Study ID (s)	Article authors and year [Citation]	Species codes*	Ocean/Region	Study site	DSC range (mg/cm^2^/day)	Exposure duration*	Coral responses*	
							Continuous	Binary
DS40	Abdel-Salam 1989, Chapter 3 [107]	8 spp.	Atlantic/Caribbean	Field	59.4	Short/Long	R, P, P/R	MO, B
DS01	Babcock and Davies 1991 [54]	AMIL	Pacific/Great Barrier Reef (GBR)	Lab	0.5—325	Short	SE	–
DS02	Babcock and Smith 2000 [25]	AMIL	Pacific/W. Australia	Field	0.7—12	Long	JS, SE	–
DS03	Bessell-Browne et al. 2017a [75]	AMIL, PORI, TREN	Pacific/GBR	Lab	0—40	Short	CHL, CI, MQY	AM, M, TM
DS48	Bessell-Browne et al. 2017b [108]	PORI	Pacific/GBR	Lab	0—20	Long	–	M
DS49	Coffroth 1985 [109]	PAST, PFUR	Atlantic/Caribbean	Field	5—78.9	Short	–	M
DS04	Duckworth et al. 2017 [110]	AMIL, MCAPI, TREN, PORI	Pacific/GBR	Lab	0.5—235	Short/Long	MQY	AM, B, TM
DS37	Fabricius et al. 2003 [59]	AWIL	Pacific/GBR	Lab	1—20.3	Short	JS	–
DS68	Flores et al. 2012 [48]	MAEQ, AMIL	Pacific/GBR	Lab	0.4—83	Long	AM, CHL, G, MPY, TM	–
DS69	Gil et al. 2016 [111]	APUL, PRUS	Pacific/French Polynesia	Lab	0.4—83	Long	TM,G	–
DS38	Goh and Lee 2008 [27]	PDAM	Pacific/Malacca Strait	Lab	0—2.5	Long	SE	–
DS42	Gowan et al. 2014 [112]	PORI	Pacific/French Polynesia	Field/ Lab	3.8—12; 17—23	Short/Long	B, G	–
DS71	HDR EOC and CSA Ocean Services 2014 [113]	PCYC, PLUT, PRUS, PCAC	Pacific/Marianas Islands	Lab	50—400	Long	TM	B, M
DS05	Hodel 2007 [76]	ACER	Atlantic/Florida	Lab	0—200	Long	-	B, M, MO, TM
DS06	Hodgson 1990b [58]	PDAM	Pacific/S. China Sea	Lab	0—1053	Long	SE	–
DS07	Hodgson 1990a [114]	OGLA, MVER, PLOB, PMEA	Pacific/S. China Sea	Lab	30—40	Short/Long	TM	AM, B
DS08 a, b	Hodgson 1989, ‘Sediment Resistance Hierarchy’ experiment [115]	36 spp., 22 spp.	Pacific/S. China Sea	Field	0—40	Short/Long	AM, TM	–
DS10	Junjie et al. 2014 [116]	GFAS, GSOM	Pacific/Singapore	Lab	26	Long	NP, MQY, P/R, R	–
DS11	Lirman et al. 2008 [117]	PAST, SSID	Atlantic/Florida	Lab	53	Long	G	AM
DS12	Loiola et al. 2013 [118]	MBRA	Atlantic/Brazil	Lab	0—450	Long	MO,PE, SI, TM	AM
DS13	Moeller et al. 2017 [87]	LPUR, AHYA	Pacific/Marianas	Field/Lab	0—1000	Long	JS	–
DS15	Perez et al. 2014 [119]	PDAM	Pacific/Hawaiʻi	Lab	0—1.5	Long	SE	
DS43	Peters and Pilson 1985 [120]	ADAN	Atlantic/Eastern US coast	Lab	0—200	Long	G, NO	AM, B, M, P/R, TM
DS16	Philipp and Fabricius 2003 [65]	13 spp.	Pacific/GBR	Field/Lab	0—200	Short	CHL, EQY, MPY, SY	AM, B, M, TM
DS17	Piniak 2007 [73]	MCAPI, PLOB	Pacific/Hawaiʻi	Lab	0—509	Short	MPY	AM, B, TM
DS18	Piniak and Brown 2008 [121]	PDAM	Pacific/Hawaiʻi	Field	38—426	Long	AM, G, TM	–
DS19	Ricardo et al. 2017 [56]	AMIL	Pacific/GBR	Lab	0—180; 0—300	Short	SE	–
DS20	Riegl and Branch 1995 [21]	FFAV, FPEN, PDAE, GINT	Indian/SW Indian Ocean	Lab	0—200	Short	M, P, R	P/R
DS21	Rogers 1979 [68]	ACER	Atlantic/Caribbean	Field	0—200	Long	G	AM, B, TM
DS45	Rogers 1983 [69]	APAL, OANN, ACER, DSTR, DCLI	Atlantic/Caribbean	Field	0—800	Short/Long	–	AM, B, TM
DS23	Selim 2007 [122]	ATEN, SPIS, PDAM	Indian/Red Sea	Lab	0—30	Short	M, SY	–
DS24	Sheridan et al. 2014 [123]	MPAT	Pacific/Madagascar	Lab	62	Short	L, ME, NO, PH, P/R	–
DS25	Shore-Maggio et al. 2018 [124]	MCAPI	Pacific/Hawaiʻi	Lab	100	Long	AM	TM
DS26	Sofonia 2006, Chapter 3 [125]	TMES, MDIG	Pacific/GBR	Lab	0—246	Long	CHL, G, L	AM, B
DS27	Sofonia 2006, Chapter 4 [125]	AFOR, MTUB, PCYC	Pacific/GBR	Field	1—372	Long	B	M, MO, TM
DS28	Sofonia and Anthony 2008 [126]	TMES	Pacific/GBR	Lab	0—12	Long	G, L, MPY	AM
DS29	Stafford-Smith 1990, Chapter 4 [127]	10 spp.	Pacific/GBR	Field	0—400	Long	AM, B, TM	TI
DS30	Stafford-Smith 1992 [128]	LPHR	Pacific/GBR	Lab	0—800	Short/Long	–	AM, B, TM
DS46	Stafford-Smith 1993 [20]	22 spp.	Pacific/GBR	Field	200	Short	–	AM, B
DS31	Stafford-Smith and Ormond 1992 [129]	42 spp.	Pacific/GBR	Field	0—50	Long	–	M, MO
DS32	Stewart et al. 2006 [71]	AHYA, PVER	Pacific/French Polynesia	Field/Lab	62.5—125	Long	SR	AM, B
DS33	Vargas-Angel et al. 2006 [72]	MCAV	Atlantic/Florida	Lab	200—225	Long	–	AM, B, M, MO, TM
DS34	Weber et al. 2006 [90]	MPEL	Pacific/GBR	Field/ Lab	33—160	Short	MQY	M, MO
DS36	Zill et al. 2017 [130]	PORI	Pacific/French Polynesia	Field	54.2	Long	G, SR	AM

*Keys to species codes and coral responses in Additional file 4. Species codes are listed when 5 species (spp.) or fewer are in study. Exposure duration: ‘short’ < 1 week, ‘long’ ≥ 1 week. Coral response is either “Continuous” data or “Binary,” indicating response data from which LOAELs and NOAELs were derived

**Table 4:** All articles and studies included in the meta-analysis of the effects of suspended sediment (SSC) on corals

Study ID (s)	Article authors and year [Citation]	Species codes*	Ocean/Region	Study site	SSC range (mg/L)	Exposure duration*	Coral responses*	
							Continuous	Binary
SS01	Anthony 1999 [131]	GRET, PCYL	Pacific/Great Barrier Reef (GBR)	Lab	0.7—16	Long	G	G
SS03 a, b	Anthony and Fabricius 2000 [78]	GRET, PCYL	Pacific/GBR	Lab	0.68—30.05	Short/Long	G, P, P/R	G, AH
SS27	Anthony et al. 2007 [74]	AINT	Pacific/GBR	Lab	0.2—10.2	Short/Long	AM, CHL, L	B, AM
SS04	Bessell-Browne et al. 2017c [132]	AMIL, MCAP, PORT	Pacific/GBR	Lab	1.17—91.69	Long	TM, AM	AM, B, TM, PE, MQY
SS05	Browne et al. 2014 [133]	MAMP, PSPE, PSIN	Indo-Pacific/Malacca Strait	Lab	0.00—242.5	Short	P/R, PE, R, NP	P/R, CHL, PE
SS06	Browne et al. 2015 [134]	MAMP, PSPE, PSIN	Indo-Pacific/Malacca Strait	Lab	1.0—92.4	Long	MPY, NP, R, TM, P/R	P/R, PE, TM, AM
SS28	Dallmeyer et al. 1982 [82]	OANN	Atlantic/Jamaica	Lab	0—525	Short	R, P	B
SS07	Erftemeijer et al. 2012b [135]	PLAC	Indo-Pacific/Singapore	Lab	6—169	Short	FS	FS
SS08	Flores et al. 2012 [48]	MAEQ, AMIL	Pacific/GBR	Lab	0—98.2	Long	TM, AM, PE, CHL, G	TM, AM, PE, CHL, G
SS11 a, b, c	Gilmour 1999 [23]	ADIG	Indian/Coastal NW Australia	Field/Lab	1.66—124.01	Short	FS, LS, LE	FS, LS, LE
SS12 a, b	Humanes et al. 2017a [81]	ATEN, AMIL, PACU	Pacific/GBR	Lab	0—100	Long	JS, G, PE, R, NP	PE, G, AM, P/R
SS13 a, b, c, d	Humanes et al. 2017b [136]	ATEN	Pacific/GBR	Lab	0.1—110.7	Short	LS, SE, FS	LS, SE, FS
SS14 a, b, c	Humphrey et al. 2008 [49]	AMIL	Pacific/GBR	Lab	0—1024	Short	FS, MO	FS, MO
SS15	Jokiel et al. 2014 [137]	PCOM	Pacific/Hawaiʻi	Field	3.1—36.8	Long	G, AM, TM, SE	-
SS16 a, b, c	Kendall et al. 1985 [138]	ACER	Atlantic/Florida	Field	0—100	Short	CAL, PRO	HI, HY, M, TE
SS17 a, b	Liu et al. 2015 [139]	AMUR	Pacific/Taiwan and Coastal China	Lab	0—45	Long	PE, CHL, SY	PE, B
SS19 a, b	Ricardo et al. 2015 [50]	ATEN, AMIL	Pacific/GBR	Lab	0—705	Short	FS	FS
SS20 a, b, c, d	Ricardo et al. 2016 [52]	AMIL, ATEN	Pacific/BR	Lab	0—1159	Short	LS	LS
SS21 a, b	Ricardo et al. 2018 [24]	AMIL, ATEN	Pacific/GBR	Lab	0—965	Short	FS	FS
SS22 a, b, c	Rice 1984 [140]	8 spp.	Atlantic/Bahamas and Florida Keys	Lab	0—199	Short/Long	G, AM	G, AM
SS24 a, b	Te 1992 [55]	PDAM	Pacific/Guam	Lab	0—1000	Long	SR	SR
SS25	Te 2001 [77]	MVER	Pacific/Hawaiʻi	Lab	27—121	Long	G, AM	P/R, G, AM, B, TM

*Keys to species codes and coral responses in Additional file 4. Species codes are listed when 5 species (spp.) or fewer in study. Exposure duration: ‘short’ < 1 week, ‘long’ ≥ 1 week. Coral response is either “Continuous” data or “Binary,” indicating response data from which LOAELs and NOAELs were derived

### Narrative synthesis including study validity assessment

This narrative synthesis was based on 86 studies from 65 articles (Tables 3, 4). Descriptive meta-data and quantitative data extracted from these studies, including study location and other effect modifiers, can be found in Additional file 3.

#### Population and exposure

For the 45 studies that explored the effects of deposited sediment on corals, there were 113 species from 53 genera. The most commonly studied genera were *Acropora* spp. (42.2%, 19 of 45), *Porites* spp. (40.0%, 18 of 45), *Montipora* spp. (31.1%, 14 of 45), and *Pocillopora* spp. (22.2%, 10 of 45). The most common species were massive *Porites lobata*/*lutea* (26.7%, 12 of 45), *Acropora millepora*, *Galaxea fascicularis*, and *Pocillopora damicornis* (each 13.3%, 6 of 45).

For the 42 studies that explored the effects of suspended sediment on corals, there were 29 species from 13 genera. The most commonly used genera were *Acropora* spp. (66.7%, 28 of 42), *Montipora* spp., *Pocillopora* spp., and *Porites* spp. (each 9.5%, 4 of 42). The most common species were *Acropora millepora* (26.7%, 12 of 42), *Acropora tenuis* (21.4%, 9 of 42), *Acropora cervicornis*, and *Acropora digitifera* (both 7.1%, 3 of 42).

Many studies were conducted using coral adults, including 37 of 45 (82.2%) of deposited sediment studies and 18 of 42 (42.9%) of suspended sediment studies. Juvenile corals were much less well represented, with only 3 of 45 (6.7%) of deposited sediment studies and 2 of 42 (4.8%) of suspended sediment studies. Studies with coral larvae were more common, with 6 of 45 (13.3%) of deposited sediment studies and 11 of 42 (26.2%) of suspended sediment studies. Coral gametes were included only for suspended sediment studies, representing 10 of 42 (23.8%) of those.

#### Comparator and study design

The design of all studies included in the synthesis were manipulative experiments, either in the field or laboratory, as described in the Methods. Manipulative experiments conducted in the laboratory represented the majority of deposited (73.3%, 33 of 45) and suspended (81.0%, 34 of 42) sediment studies. The remainder were conducted in the field, or in lab-based mesocosms that mimicked field conditions. Additionally, all studies included in the synthesis experimentally exposed corals to sediment and included appropriate experimental controls. In the lab, the control corals were exposed to no or extremely low levels of sediment, while in the field, the control corals were exposed to non-augmented, ambient levels of sediment.

#### Outcomes

Corals experience adverse effects in response to sediment stress through a variety of biological mechanisms. Below is a review of these mechanisms, organized by coral response and life-history stage.

*Reduced fertilization of coral gametes* Many possible cause-effect pathways may link early life-history stages of corals with sediment stress, yet these remain largely untested [28]. In particular, sediments may negatively affect gamete viability or obstruct egg–sperm contact [28, 49, 50], leading to reduced fertilization success, thereby reducing the chance of successful recruitment, population maintenance, and recovery. Ricardo et al. [50] revealed that fine, siliciclastic sediments cause sediment–sperm flocs, resulting in fewer available sperm to fertilize buoyant eggs. The biogeochemical mechanism by which coral sperm adhere and are stripped from the water surface in sinking flocs remains unclear.

*Mortality of coral larvae* Suspended sediment may reduce larval survival through decreased light availability and intensity [18] and physical abrasion [23]. Suspended sediment increases light attenuation, decreasing light availability in the water column. Planktonic coral larvae feed and receive translocated metabolites from their zooxanthellae [51]. Decreased photosynthetic efficiency of larval symbionts from low light levels for extended periods of time may lead to larval mortality from starvation. There is evidence that mucus secretion and cilia beating protects planktonic coral larvae from suspended sediment after 60 h of exposure [52].

*Reduced settlement of coral larvae* Increased light attenuation due to suspended sediment may decrease larval settlement because light quality and quantity are factors in site selection for coral larvae. Coral larvae may preferentially settle on the top of surfaces in low light levels [53]. Settling on exposed upper surfaces increases the risk of abrasion and burial of corals by suspended and deposited sediment, which could lead to low recruit survival. Larvae avoid abrasion and smothering in the presence of sediment when they settle on downward facing surfaces [54]. Larvae that settle in highly turbid areas that are less suitable for survival may undergo reversed metamorphosis and revert back to a swimming larva [55].

Sediment cover on the benthos can prevent larvae from sensing chemical or textural cues that induce settlement [56, 57], including altered bacterial cues [27]. Decreased coral settlement on sediment-covered surfaces has been previously observed for *Pocillopora damicornis* [58], *Acropora digitifera* [23], and *Acropora millepora* [56].

*Mortality of coral recruits* Settlement of coral larvae onto exposed, vertically facing surfaces increases the risk of abrasion and burial by suspended and deposited sediment, which may reduce their survival as juvenile recruits. Fabricius et al. [59] found that recruits were one to two orders of magnitude more sensitive to sedimentation than adult corals. The coral polyps of recruits may be smothered by deposited sediment [60], the accumulation of which may prevent coral tentacles from feeding and diminish light availability for photosynthesis in symbiotic algae.

*Decrease in photosynthesis-to-respiration ratio of adult corals* The ratio of production (or photosynthesis) to respiration (P/R) is used as an indicator of coral energy budgets. A P/R ratio below 1 indicates more energy is being used than produced. P/R ratios may fluctuate throughout the day, but a low P/R for an extended period of time means corals are using energy reserves. The P/R ratio may decrease if gross photosynthesis decreases due to low light availability in turbid water, or increased respiration rates as a result of increased metabolic activity in response to suspended sediment exposure [21, 61]. A decline in productivity can lead to starvation of the coral [21]. Abdel-Salam and Porter [62] observed decreased gross photosynthesis and increased respiration in corals smothered by sediment, leading to decreased P/R ratios.

*Reduced photosynthetic efficiency of adult corals* Pulse Amplitude Modulation (PAM) fluorometry is often used to measure the photosynthetic efficiency of Photosystem II of coral endosymbiotic zooxanthellae. Since corals rely on symbionts for up to 90% of their energy [63], a decrease in their photosynthetic efficiency is used as an indicator of decreased energy availability for corals. Measurements in the literature are most often “quantum yield” (*F*_*v*_*/F*_*m*_), a decrease in which is believed to be an early sign of coral bleaching [64] and is often used as an indicator of health of the coral symbiont, and thus of the host coral. Declines in photosynthetic efficiency may result from physical damage of coral tissue and its symbionts due to shearing in turbid conditions, or from deposited sediment on the coral. Philipp and Fabricius [65] observed decreases in quantum yield in corals exposed to sediment, but only in areas that accumulated sediment on the tissue. Symbionts can often recover, but recovery depends on the duration and concentration of sediment exposure [65].

*Bleaching of adult corals* Large-scale coral bleaching is most strongly related to increased temperatures and irradiance levels [66, 67], but there is evidence of sediment-induced bleaching [16, 65, 68–73]. Deposited and suspended sediment often result in a reduced energy state for the coral due to light attenuation and the shift in energy allocation to sediment removal [48, 74]. This reduced energy state can leave corals sensitive to bleaching and may induce symbiont expulsion after prolonged sediment exposure [75]. Bleaching is often a precursor to tissue mortality due to the accompanying stressors of deposited and suspended sediments (i.e., starvation, hypoxia, abrasion, microbially mediated tissue damage, and tissue irritation) [65, 70, 72, 76]. However, there is some evidence that high turbidity can lead to lower susceptibility of bleaching due to shadowing when temperature is a covariate [74, 77].

*Decreased growth rate of adult corals* The biological mechanisms driving the growth responses in corals are related to energy allocation and availability [78]. High levels of suspended sediment result in light attenuation forcing corals to compensate via increased pigmentation or symbiont densities or by shifting nutrient acquisition to more dependence on heterotrophy [78]. Colonies that are unable to acclimate may respond similarly to those in shaded conditions, resulting in much lower skeletal growth rates and thinner tissues due to decreased energy investment in growth and accretion [79]. Increased turbidity and deposited sediment can also result in irritation and abrasion of coral tissue, especially if paired with wave action. This, too, may result in an energy budget with more resources put towards survival than growth. Deposited sediments also affect energy expenditures due to disruptions in feeding mechanisms (e.g., production of mucus cords) and may shift energy allocation towards self-cleaning through increased tentacle movement and mucus production [18, 21, 80, 81].

Generally, growth rates are negatively affected by both suspended and deposited sediment, but the magnitude of the decrease is dependent on other factors or life-history strategies [78], including coral morphology, species, level of heterotrophic dependency, and sediment composition [17, 48, 81]. Interestingly, the differences in response may ultimately lead to selection of coral communities composed of more branching morphologies in high sedimentation environments because these branching forms shed sediment more readily than other forms (e.g., massive and plating).

*Mortality of adult corals* Mechanisms that mediate partial and/or total tissue mortality of adult corals in response to sediment exposure include light inhibition [68, 74], smothering [60], increased energy allocation to sediment clearance [62, 82], and tissue damage [83]. Suspended sediment decreases light availability to corals, leading to a decrease in gross photosynthesis. During periods of low light, corals can use heterotrophic feeding to meet their energetic demands, but heterotrophic feeding decreases when polyps retract in response to deposited sediment. A decline in autotrophic energy production of coral symbionts paired with an inability to enhance heterotrophic feeding may lead to coral starvation. Prolonged periods of sediment sloughing by increased mucus production may also deplete coral energy reserves. Dead patches under sediment occur when sloughing of sediment is no longer possible [48, 65, 84]. If deposited sediment is nutrient-rich, it could enhance microbial growth and lead to flocculation of sediment [60]. Long periods of increased sediment exposure, or more frequent exposure events, have been shown to cause coral mortality [85] and may lead to permanent changes in coral-reef community structure as some species adapt to high-sediment environments and others do not [86].

**Fig. 4:**
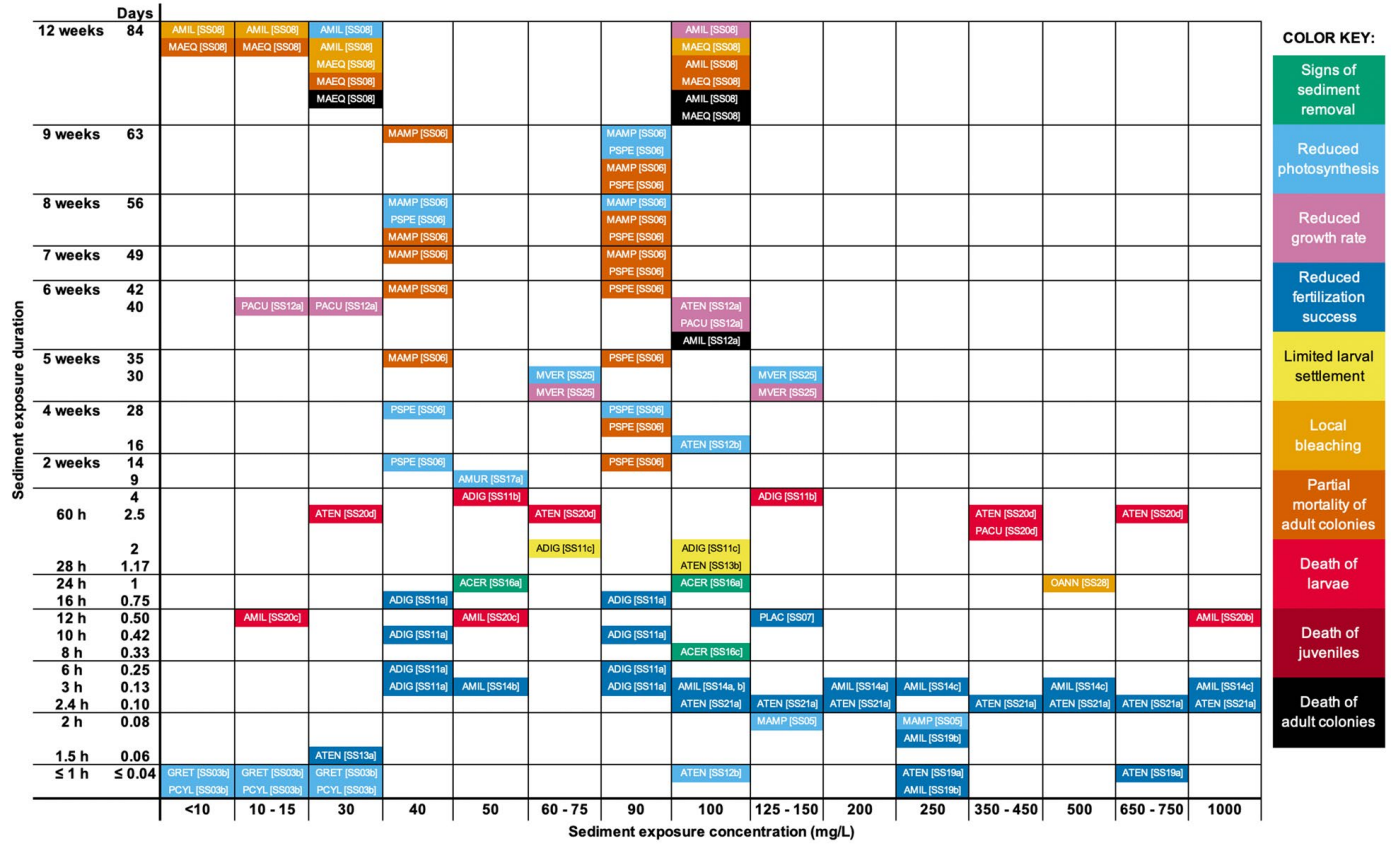
Review of coral responses to varying suspended sediment concentrations at timescales ranging from minutes to months. Coral responses are color coded with a key shown at the right of this figure. Coral species are shown as four-letter codes, with a key provided at Additional file 4. Key to numbered references [SS##] are provided at Table 4

**Fig. 5:**
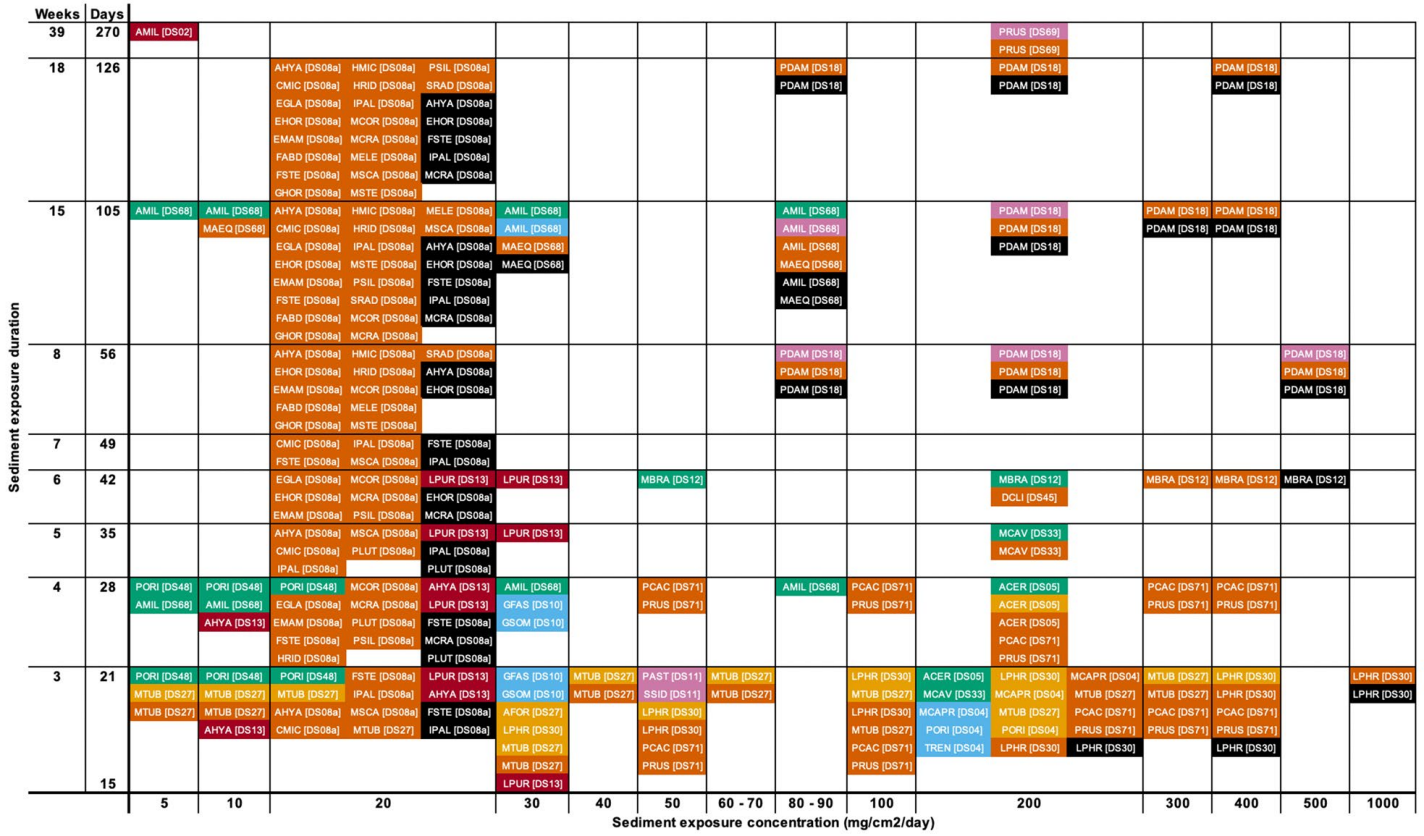
Review of coral responses to varying deposited sediment concentrations at timescales ranging from 15 to 270 days (> 2 weeks to 39 weeks). Coral responses are color coded with a key shown at the right side of Fig. 4. Coral species are shown as four-letter codes, with a key provided at Additional file 4. Key to numbered references [DS##] are provided at Table 3

**Fig. 6:**
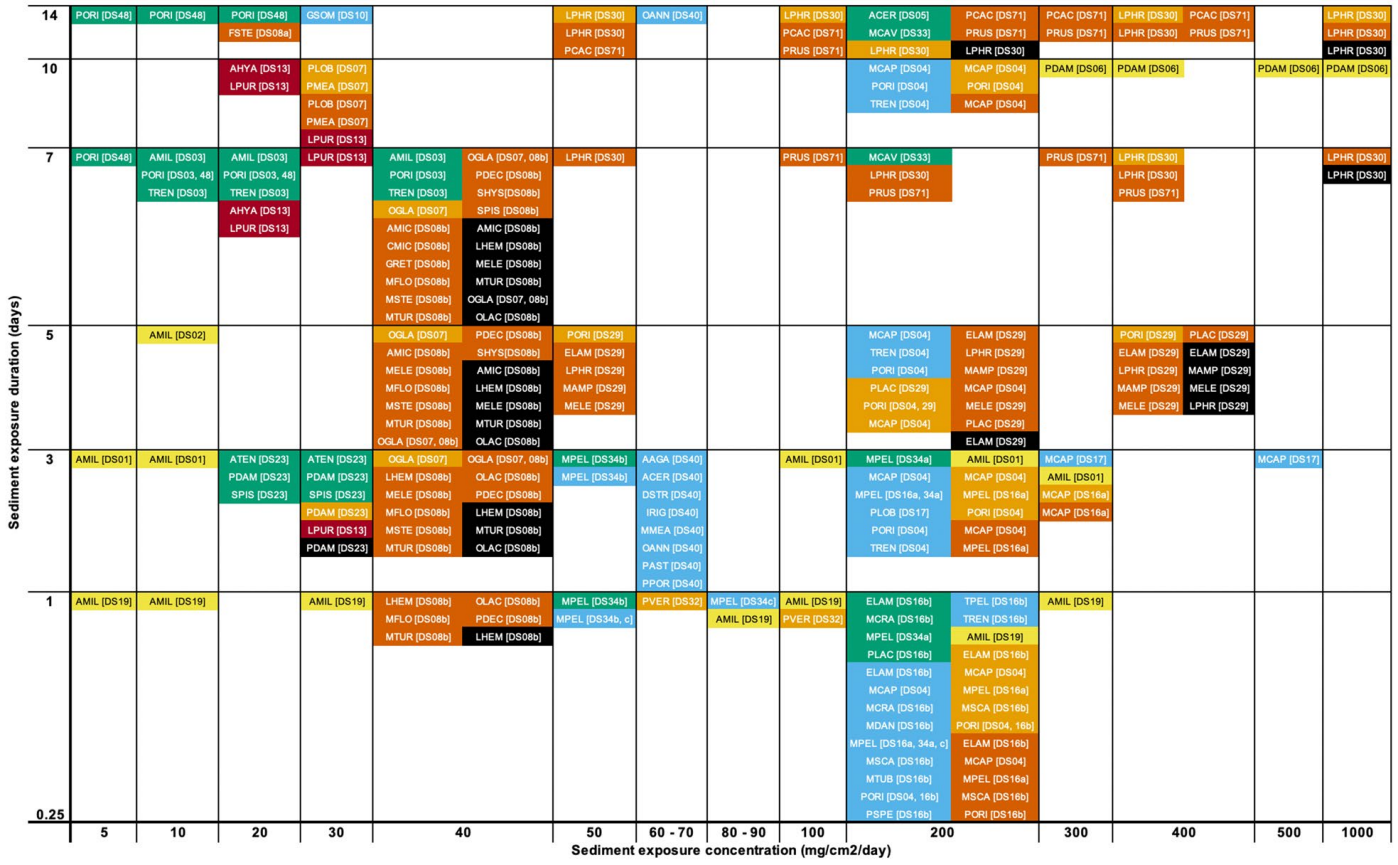
Review of coral responses to varying deposited sediment concentrations at timescales ranging from hours to 2 weeks. Coral responses are color coded with a key shown at the right side of Fig. 4. Coral species are shown as four-letter codes, with a key provided at Additional file 4. Key to numbered references [DS##] are provided at Table 3

To display the range of biological mechanisms and coral responses to sediment exposure, we coded all reported coral responses by species and study and show corresponding concentrations and durations of sediment exposure (Figs. 4, 5, 6). We categorize responses into immediate, short-term, and medium-to-long-term:

- Immediate responses include behavioral changes that remove sediment from the coral’s surface and are rarely considered adverse, unless the behaviors persist for long enough to significantly diminish a coral’s energy reserves. *Examples* Hydrostatic inflation, movement of tentacles, and increased mucus production and sloughing (green ‘Signs of Sediment Removal’ in Figs. 4, 5, 6).
- Short-term responses include physiological changes that are likely adverse if they persist. Some short-term responses can be lethal if experienced by coral gametes or larvae, which spend relatively little time in these developmental stages (hours to a few days). *Examples* Reduced photosynthesis (in terms of photosynthetic efficiency or ratios of photosynthesis-to-respiration; light blue in Figs. 4, 5, 6), localized bleaching (light orange in Figs. 4, 5, 6), and reduced fertilization success of gametes (dark blue in Figs. 4, 5, 6). Larvae experience limited settlement rates (yellow in Figs. 4, 5, 6) and pre-settlement mortality (red in Figs. 4, 5, 6).
- Medium- to long-term responses are usually considered adverse and are often lethal at the scale of the individual coral polyp or whole colony. *Examples* Coral adults experience reduced growth rate (mauve in Figs. 4, 5, 6), tissue necrosis (orange in in Figs. 4, 5, 6), and colony mortality (black in in Figs. 4, 5, 6). Juveniles can also experience mortality (dark red in Figs. 4, 5, 6), and thus reduced recruitment rates.

Other less commonly reported responses of corals to sediment stress are listed in Additional file 4, along with a key to the coral species codes used in Figs. 4, 5, 6.

#### Study validity assessment

Factors that may affect the validity of each study, including study setting and temporal extent, are found in Tables 3 and 4 for all studies that were included in the review (both the narrative and data syntheses). Other factors that may influence external and internal validities of studies were used to assess for the presence of selection, performance, attrition, reporting, and other miscellaneous biases and given an overall environmental validity GRADE—very low, low, moderate, or high—based on these assessments [Additional file 3]. Of the 44 articles included in the review of the effects of deposited sediment on corals, none were given a ‘very low’ or ‘low’ GRADE, 20.5% (9 of 44) a ‘moderate’ GRADE, and 79.5% (35 of 44) a ‘high’ GRADE. Of the 22 articles included in the review of the effects of suspended sediment on corals, none were given a ‘very low’ or ‘low’ GRADE, 45.5% (10 of 22) a ‘moderate’ GRADE, and 54.5% (12 of 22) a ‘high’ GRADE. No articles were given low GRADEs at this stage, presumably because articles and studies with high levels of bias (and thus a low overall GRADE) were screened out at the full-text review stage when the PECO framework was used to identify and remove articles with inappropriate populations, exposures, comparators, outcomes, and study designs [see Additional file 3 for reasons for exclusion]. An article received a moderate GRADE when there was unclear evidence for two or more types of bias. The two types of bias that most commonly resulted in a moderate GRADE were reporting bias (difficulty discerning whether a study had reported all of the findings from its pre-specified primary outcomes) and selection bias (lack of detail on the randomization process used to assign coral specimens to treatments). Articles with moderate and high GRADEs were included in the review and analyses.

### Data synthesis

#### Description of the data

All 86 studies from 65 articles that we included in the narrative synthesis were further included in our data synthesis database [Additional file 3]. Of these studies, 65.1% were assessed as having ‘high’ overall validity and 34.9% as having ‘moderate’ overall validity. There were no studies with ‘low’ overall validity, which is most likely the result of the PECO framework identifying relatively low-quality studies that were subsequently removed at the full-text screening stage.

#### LOAELs and NOAELs

**Fig. 7:**
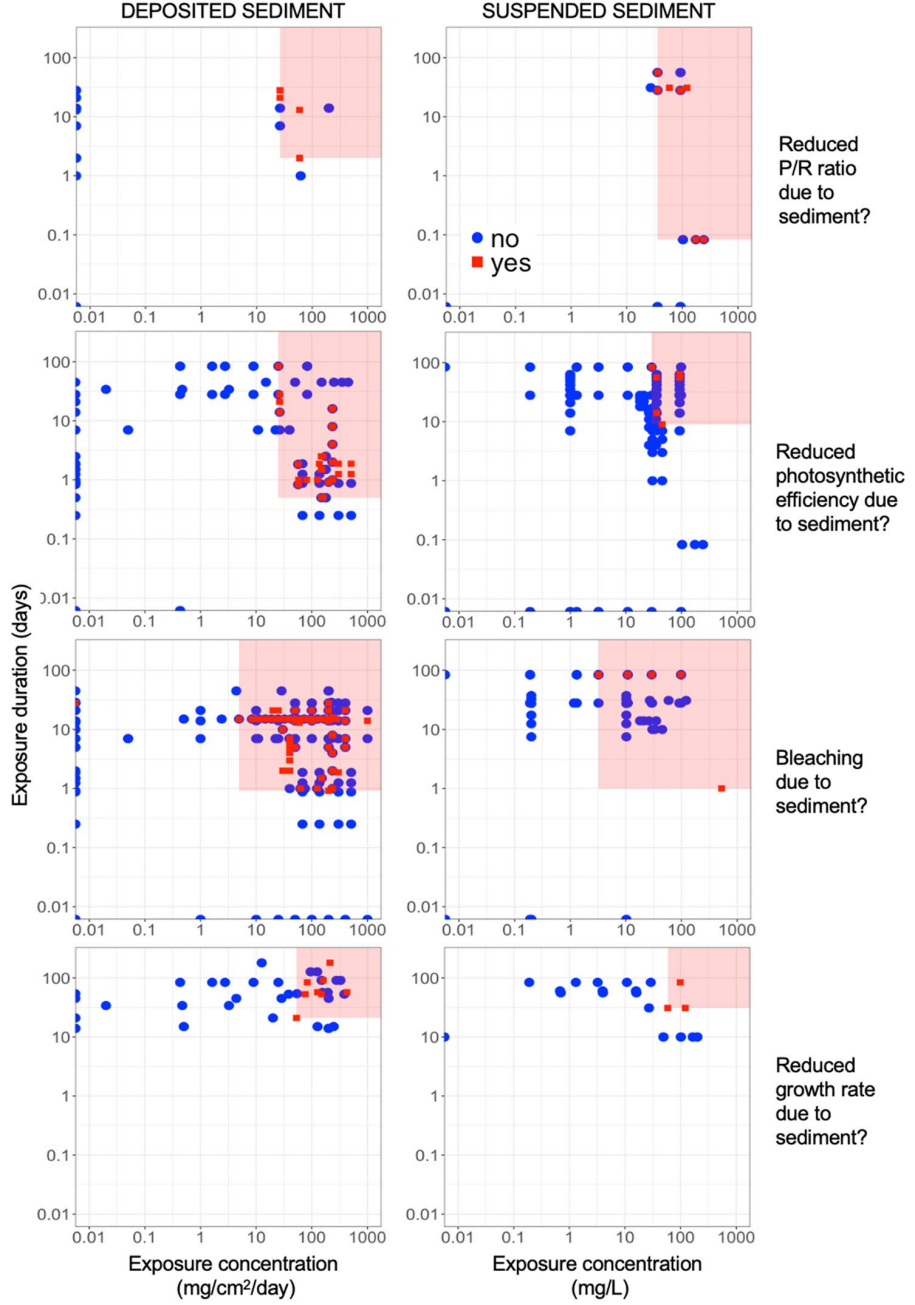
Detection of adverse effects for physiological effects of sediment exposure on coral adults, plotted as concentration vs. duration of exposure to either deposited sediment (left panels) or suspended sediment (right panels). Each row of panels represents a different coral response. The red, rectangular area is bounded by the LOAELs for concentration and duration, thereby representing the exposure conditions under which adverse effects have been observed in studies from our review

**Fig. 8:**
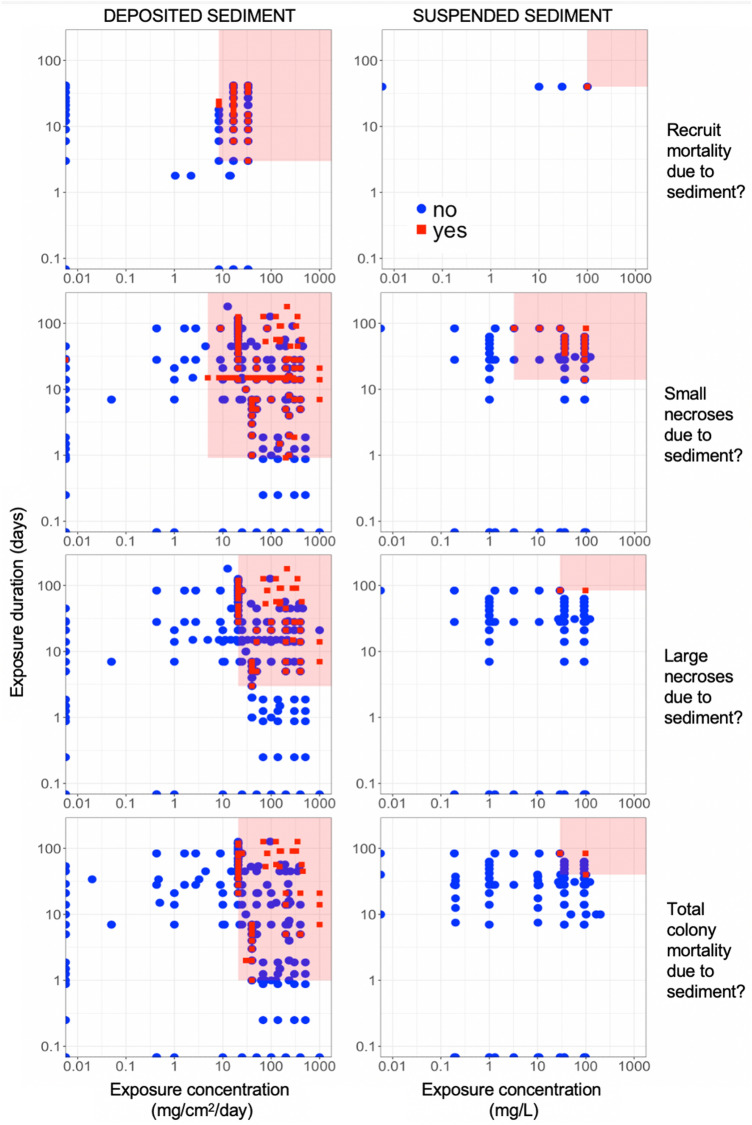
Detection of adverse effects for lethal effects of sediment exposure on coral juveniles and adults, plotted as concentration vs. duration of exposure to either deposited sediment (left panels) or suspended sediment (right panels). Each row of panels represents a different coral response. Small necroses are < 50% of adult coral tissue area, large necroses are ≥ 50% and < 100% tissue area, and both recruit and total mortality are 100% tissue necrosis. The red, rectangular area is bounded by the LOAELs for concentration and duration, thereby representing the exposure conditions under which adverse effects have been observed in studies from our review

Inclusive of all coral developmental stages, taxa, and geographic origins, deposited sediment concentrations (DSC) as low as 1 mg/cm^2^/day and suspended sediment concentrations (SSC) as low as 3.2 mg/L can adversely affect corals (LOAELs; Tables 5, 6). Physiological responses (e.g., reduced photosynthesis of symbionts) can occur as quickly as 12 h and 1 h after exposure to deposited sediment and suspended sediment, respectively (Tables 5, 6; Fig. 7). Lethal responses (i.e., tissue necrosis) occur at DSC as low as 4.9 mg/cm^2^/day and for exposure durations less than one day (22 h) (Table 5, Fig. 8). Lethal responses can occur after exposure to SSC as low as 3.2 mg/L and 12 h.

**Table 5:** Coral response thresholds (NOAEL/LOAEL) of all studies concerning the effects of deposited sediment on corals

Coral age class	Binary coral response		# Treatment groups* (controls included/excluded)		# Studies/articles with binary data		# Species/genera with binary data		NOAEL				LOAEL			
									Concentration (mg/cm^2^/day)		Duration		Concentration (mg/cm^2^/day)		Duration	
Gametes	Reduced fertilization success?		–		–		–		–		–		–		–	
Larvae	Larval mortality?		–		–		–		–		–		–		–	
	Limited settlement?		54/45		4/4		2/2		1.0		–		1.0		–	
Juveniles	Recruit mortality?		132/87		3/3		4/2		8.3		3 days		8.3		3 days	
Adults	Reduced P/R ratio?		60/25		5/5		16/15		26.4		2 days		26.4		2 days	
	Reduced photosynthetic efficiency?		372/249		9/9		20/12		25.0		12 h		25.0		12 h	
	Local bleaching?		497/352		20/20		52/32		4.9		22 h		4.9		22 h	
	Reduced growth rate?		55/40		10/10		10/7		38.4		21 days		53.0		21 days	
	Small tissue necroses?		750/602		21/20		76/39		4.4		22 h		4.9		22 h	
	Large tissue necroses?		657/522		17/17		75/39		20.8		3 days		20.8		3 days	
	Total colony mortality?		678/509		24/23		84/46		20.8		1 day		20.8		1 day	
**Adults**	ANY MORTALITY?*		827/629		28/27		87/46		4.4		22 h		4.9		22 h	
	ANY ADVERSE EFFECT?*		1085/783		34/34		101/50		4.9		12 h		4.9		12 h	
**All**	ANY MORTALITY?*		965/719		31/30		89/47		4.4		22 h		4.9		22 h	
	ANY ADVERSE EFFECT?*		1323/943		40/39		102/51		1.0		12 h		1.0		12 h	

*A ‘*treatment group*’ is an experimental unit of corals exposed to the same exposure conditions within a study—these may be control (no sediment exposure) or treatment conditions of differing exposure concentrations and/or durations. Double dashes ‘–’ indicate that data were non-existent or irrelevant. *Any adverse effect* is defined as any response of a coral individual, colony, or treatment group that may negatively affect a coral’s fitness and/or survival. These adverse effects may include physiological changes (e.g., reduced growth or photosynthetic rates), bleaching, tissue necrosis, and colony mortality. *Any mortality* is inclusive of death of tissue (small and large necroses) or of the entire coral colony, and thus excludes sublethal coral responses

**Table 6:** Coral response thresholds (NOAEL/LOAEL) of all studies concerning the effects of suspended sediment on corals

Coral age class	Binary coral response	# Treatment groups* (controls included/excluded)	# Studies/articles with binary data	# Species/genera with binary data	NOAEL threshold		LOAEL threshold	
					Concentration (mg/L)	Duration	Concentration (mg/L)	Duration
Gametes	Reduced fertilization success?	110/86	10/6	4/2	25.0	–	30.4	–
Larvae	Larval mortality?	63/52	7/4	5/2	29.5	–	30.0	–
	Limited settlement?	30/20	7/4	4/3	34.6	–	57.8	–
Juveniles	Physiological limitation?	20/15	2/1	3/2	10.0	0	10.0	1 h
	Recruit mortality?	16/9	2/2	4/3	100.0	40 days	100.0	40 days
Adults	Reduced P/R ratio?	49/34	3/3	4/4	35.8	2 h	35.8	2 h
	Reduced photosynthetic efficiency?	238/180	6/5	8/6	35.8	56 days	35.8	56 days
	Local bleaching?	92/54	8/7	10/6	3.2	1 day	3.2	1 day
	Reduced growth rate?	79/47	7/5	12/12	49.0	31 days	58.6	31 days
	Small tissue necroses?	210/147	4/4	8/6	3.2	14 days	3.2	14 days
	Large tissue necroses?	210/147	4/4	8/6	29.1	84 days	29.1	84 days
	Total colony mortality?	272/176	8/6	17/14	29.1	40 days	29.1	40 days
Adults	ANY MORTALITY?*	272/176	8/6	17/14	3.2	14 days	3.2	14 days
	ANY ADVERSE EFFECT?*	360/244	14/11	21/16	3.2	2 h	3.2	2 h
All	ANY MORTALITY?*	376/261	19/11	21/15	3.2	12 h	3.2	12 h
	ANY ADVERSE EFFECT?*	585/423	37/20	26/18	3.2	0	3.2	1 h

*A ‘*treatment group*’ is an experimental unit of corals exposed to the same exposure conditions within a study—these may be control (no sediment exposure) or treatment conditions of differing exposure concentrations and/or durations. ‘*Physiological limitation?*’ for juvenile corals indicates either reduced P/R ratio, reduced photosynthetic efficiency, or reduced growth rate. These are combined here because they represent the physiological results from only one article. = Double dashes ‘–' indicate that data were irrelevant. *Any adverse effect* is defined as any response of a coral individual, colony, or treatment group that may negatively affect a coral’s fitness and/or survival. These adverse effects may include physiological changes (e.g., reduced growth or photosynthetic rates), bleaching, tissue necrosis, and colony mortality. *Any mortality* is inclusive of death of tissue (small and large necroses) or of the entire coral colony, and thus excludes sublethal coral responses

**Fig. 9:**
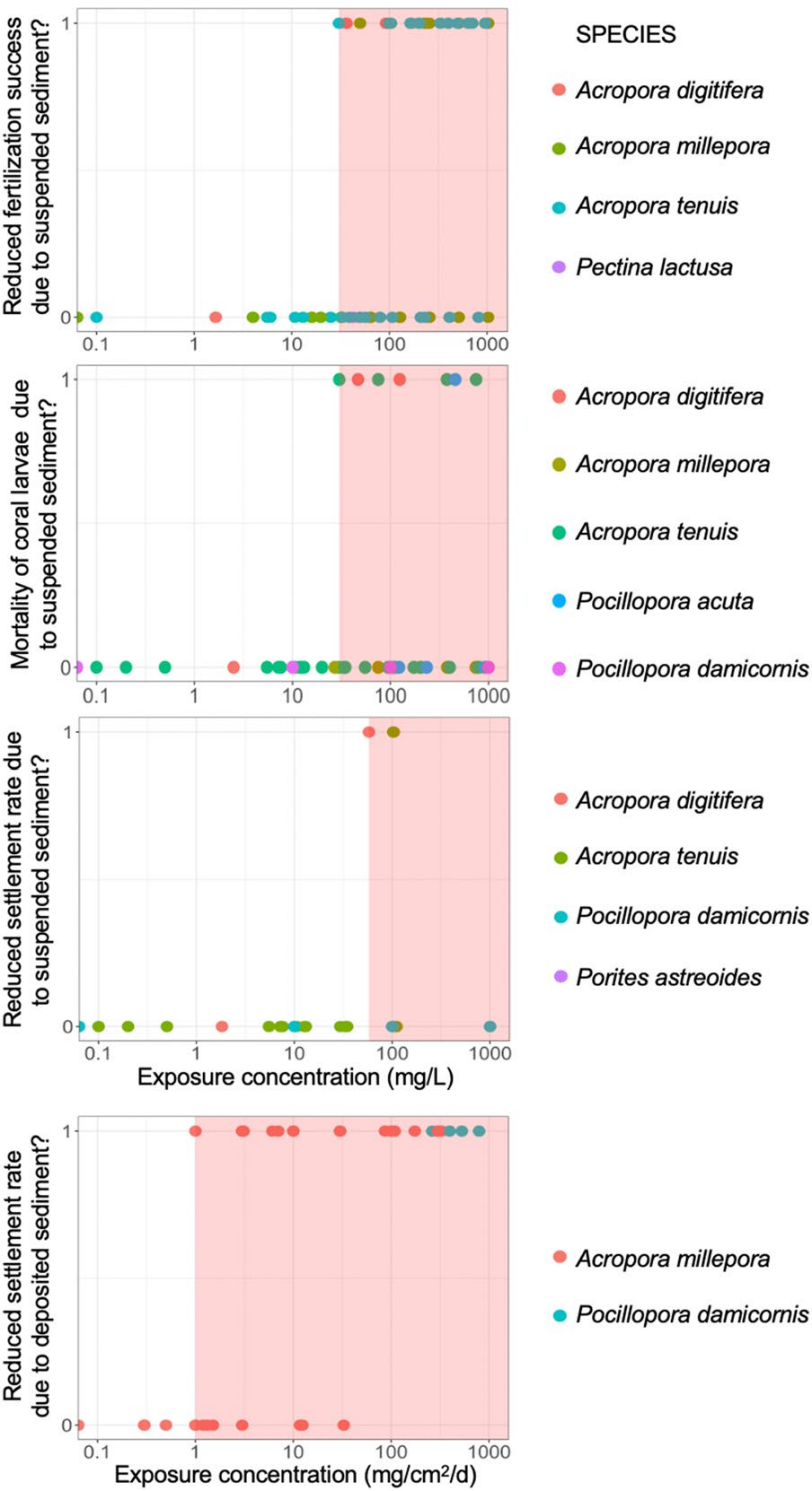
Detection of adverse effects for coral gametes and larvae in response to sediment exposure (0 = no, 1 = yes), plotted as a function of concentration of either suspended sediment (top 3 panels) or deposited sediment (bottom panel). The red, rectangular area is bounded by the concentration LOAEL, thereby representing the exposure concentration under which adverse effects have been observed in studies from our review. Given the short period of time that corals are in these life-history stages, exposure duration was not considered in the determination of NOAEL/LOAELs, nor in meta-analyses

When we consider only mature, adult corals, results are similar. However, adults are slightly less sensitive to deposited sediment than immature coral stages (cf. Figures 7, 8, 9), with adverse responses beginning to occur at 4.9 mg/cm^2^/day and after 12 h (Table 5). Adults begin to bleach at 3.2 mg/L SSC after only 2 h exposure (Table 6; Fig. 7) and experience tissue necrosis at 3.2 mg/L after at least 2 weeks (14 days) of exposure to suspended sediment (Table 6; Fig. 8). While these minimum values at which adverse effects are observed (LOAELs) in corals appear low for suspended sediment exposure, corals typically took an order of magnitude times longer to experience lethal effects due to suspended sediment than to comparable concentrations of deposited sediment (cf. Tables 5 and 6; Figs. 7, 8, 9).

#### Dose–response meta-analyses

We also used dose–response meta-regression analyses (DRMA) to model the relationship between sediment exposure and the magnitude of coral responses, where available data were sufficient. The dose–response thresholds reported below are the lowest concentrations at which sediment-exposed corals (‘treatment’) are expected to have a lower or reduced response than corals not exposed to sediment (‘controls’) (Fig. 2C). These are statistically significant differences between treatment and control corals, with 95% confidence, which may not reflect biologically significant differences in some cases. Biological significance is dictated by ecological context (i.e., species, population, location, etc.), and therefore could not be easily synthesized across studies.

**Fig. 10:**
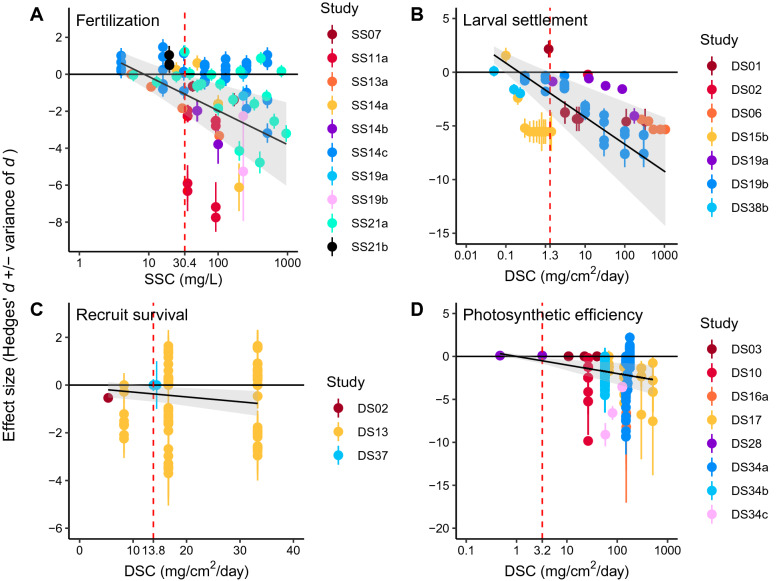
Effect sizes (Hedges’ *d* ± *s*) for fertilization success (**A**), larval settlement (**B**), recruit survival (**C**), and photosynthetic efficiency (**D**) data, by study, with the best-fit dose–response meta-regression (DRMA) model’s predictions (mean is solid, black line; 95% CI is shaded in gray) and the DRMA threshold value indicated on the x- axis, below the red, dashed line (where 95% CI no longer overlaps with zero). *DSC* deposited sediment concentration, *SSC* suspended sediment concentration

*Coral gametes* Coral gametes have significantly reduced fertilization success at 30.4 mg/L suspended sediment and greater (Table 7; Fig. 10A). This dose–response threshold matches exactly with the LOAEL in Fig. 9 (LOAEL = 30.4 mg/L, NOAEL = 25.0 mg/L). The DRMA was based on the standardized results of 10 studies from 6 articles that used 4 coral species from 2 genera: *Acropora digitifera*, *Acropora millepora*, *Acropora tenuis*, and *Pectina lactusa*, in the Indian and Pacific Oceans (Table 4). Exposure durations were brief (< 1 h) and relatively standardized across studies, so this factor was not considered in the DRMA or determination of thresholds.

The best-fit DRMA model’s I2 statistic was 82%, indicating considerable residual heterogeneity unaccounted for by the model (Table 7), which could be the result of taxonomic, geographic, and/or mineralogical differences among (and within) studies. However, model diagnostics revealed a mostly symmetrical, inverted funnel [Additional file 3: Fig. S2A], indicating little evidence for publication bias among included studies. One study (SS11a) [23] had greater effect sizes than expected given its level of precision, which may be due to fertilization success being measured after a slightly longer sediment exposure (45 min) than other studies (≤ 30 min).

**Table 7:** Results of best-fit dose–response meta-regression (DRMA) models for coral responses where sufficient data were available to assess the relationship between sediment exposure (‘dose’) and magnitude of the coral response of-interest (standardized effect size, Hedges’ *d*)

Coral age class	Continuous coral response	Deposited or suspended sediment*	# Treatment groups* (controls included/excluded)	# Studies/articles in DRMA	# Species/genera in DRMA	Dose–response I^2^ Statistic*	Dose–response threshold* (DS: mg/cm^2^/day; SS: mg/L)
Gametes	**Fertilization success rate**	**SS**	**110/86**	**10/6**	**4/2**	**82.3%**	**30.4 (*p* < 0.0001)**
Larvae	Larval survival rate	SS	50/42	4/3	4/2	73.0%	*n.s*
	**Settlement rate**	**DS**	**71/61**	**7/6**	**2/2**	**84.6%**	**1.3 (*p* < 0.0001)**
	Settlement rate	SS	26/20	6/3	3/2	88.3%	*n.s*
Juveniles	**Recruit mortality rate**	**DS**	**132/87**	**3/3**	**4/2**	**47.1%**	**13.8 (*p* = 0.025)**
Adults	P/R ratio	DS	20/10	3/3	4/4	58.4%	*n.s*
	**Photosynthetic efficiency**	**DS**	**181/141**	**8/6**	**9/6**	**76.8%**	**3.2 (*p* = 0.005)**
	Photosynthetic efficiency	SS	217/164	5/4	6/5	21.4%	*n.s*
	Growth rate	DS	29/19	8/8	8/5	41.5%	*n.s*
	Partial tissue mortality rate	DS	140/115	4/4	11/8	86.9%	*n.s*
	Total colony mortality rate	SS	47/33	4/4	6/4	0.0%	*n.s*

Analyses using deposited or suspended sediment datasets are indicated as ‘DS’ and ‘SS,’ respectively. A ‘*treatment group*’ is an experimental unit of corals exposed to the same exposure conditions within a study—these may be control conditions (no sediment exposure) or treatment conditions of differing exposure concentrations and/or durations. The ‘*Dose–Response I*^*2*^* Statistic*’ is a measure that indicates the percentage of variance in a meta-analysis that is attributable to heterogeneity among dose–response comparisons within study. Heterogeneity is substantial when I^2^ is above 75%. The ‘*Dose–Response Threshold*’ for a coral response significantly affected by sediment concentration was the minimum exposure value at which DRMA 95% CI no longer overlapped with zero (where zero indicates no difference between a treatment group and its control, see Fig. 2C). Rows in bold represent significant relationships (*p* ≤ 0.05) between sediment exposure and the effect size of the corresponding coral response and ‘*n.s.*’ indicates a non-significant relationship (*p* > 0.05)

*Coral larvae* Settlement rates of coral larvae on vertically facing surfaces (those most susceptible to sediment deposition) significantly decline at 1.3 mg/cm^2^/day deposited sediment and greater (Table 7; Fig. 10B). This dose–response threshold also closely aligns with the NOAEL and LOAEL derived in Fig. 9 (1 mg/cm^2^/day). The DRMA was based on the standardized results of 7 studies from 6 articles that investigated the effect of deposited sediment on settlement rate of larvae from 2 coral species: *Acropora millepora* and *Pocillopora damicornis*, all in the Pacific Ocean (Table 3).

The best-fit model’s I^2^ statistic was 84.6%, indicating considerable residual heterogeneity unaccounted for by the model (Table 7), which could be the result of taxonomic, geographic, and/or mineralogical differences among (and within) studies. However, model diagnostics revealed a mostly symmetrical, inverted funnel [Additional file 3: Fig. S1A], indicating little evidence for publication bias among included studies.

We found no significant relationships between suspended sediment concentrations and rates of either larval survival or settlement (Table 7). For these relationships, it is likely that suspended sediment weakly interacts with coral larvae through decreased light availability in the water column, thus affecting symbiotic algae, but that secretion of protective mucus and beating of cilia may protect the planktonic coral larvae from suspended particles after 60 h of exposure [52].

*Coral juveniles* Survival of coral juvenile recruits significantly declines at 13.8 mg/cm^2^/day deposited sediment and greater (Table 7; Fig. 10C). This is a less conservative threshold estimate than suggested by the NOAEL and LOAEL of 8.3 mg/cm^2^/day in Fig. 8. The DRMA was based on the standardized results of 3 studies from 3 articles that investigated the effect of deposited sediment on mortality of recruits from 4 coral species from 2 genera: *Acropora hyacinthus*, *A. millepora*, *Acropora willisae*, and *Leptastrea purpurea*, all in the Pacific Ocean (Table 3).

The best-fit DRMA model explained over half (53%) of the variability in juvenile survival (I^2^ = 47% residual heterogeneity). A diagnostic funnel plot indicated little evidence for publication bias [Additional file 3: Fig. S1B], though datapoints (i.e., study treatment groups) with higher levels of precision were sparse. One study represented the bulk of the data used in the model, DS13 [87], the variability from which was a factor of juvenile age (from 2 days to 8 weeks post-settlement, at start of experiment), species (*A. hyacinthus* and *L. purpurea*), and sediment exposure level (0, 16.6, and 33.3 mg/cm^2^/day).

*Coral adults* Photosynthetic efficiency (maximum quantum yield, *F*_*v*_*/F*_*m*_) significantly declines at 3.2 mg/cm^2^/day deposited sediment and greater (Table 7; Fig. 10D). This estimate is much less than the NOAEL and LOAEL of 25 mg/cm^2^/day from Fig. 7. The DRMA was based on the standardized results of 9 studies from 9 articles that investigated the effect of deposited sediment on the photosynthetic efficiency among adults of 20 species from 12 genera in 3 oceans (Table 3).

There was considerable heterogeneity unaccounted for in the DRMA model (I^2^ = 81%), which may indicate that the dose–response threshold is less robust. However, most studies that measure *F*_*v*_*/F*_*m*_ tested exposure concentrations at or above 25 mg/cm^2^/day, indicating that future studies should explore the effects of lower exposure levels before a more definitive threshold can be estimated. Regardless, a diagnostic funnel plot showed little evidence for publication bias [Additional file 3: Fig. S1D].

We found no significant relationships between deposited sedimentation rates and P/R ratio, growth rate, or partial mortality rate, nor between suspended sediment concentrations and photosynthetic efficiency or total mortality rate (Table 7). For these relationships, there is likely too much variability to detect an effect across studies. This may be due, in part, to the overwhelming taxonomic diversity represented within these studies, which included 62 species from 31 genera.

### Review limitations

The results and thresholds that we present should be interpreted within the context of the studies that were included as part of this systematic review and meta-analysis. In particular, there are limitations inherent to the design and reporting of experiments. There are also research gaps brought to light by the interpretation of certain meta-analytical models. Lastly, there are limitations inherent to our review methods. We discuss these limitations and gaps below, which represent opportunities to improve future work.

#### Limits of study design

*Scope of inference* We chose to focus on manipulative experiments so that we could directly ascribe the adverse effects experienced by corals to sediment exposure and not to other confounding variables like nutrient-enrichment, contamination, etc. Most manipulative experiments took place in the lab where sediment exposure could be precisely measured instead of in situ, where sedimentation and resuspension regularly occur. Therefore, the thresholds for sediment exposure described herein may not match apparent thresholds identified in the field or in individual experiments that focus on a limited set of taxa.

The thresholds we identify are likely to be less conservative than those experienced by corals on reefs, which face multiple stressors that may cause adverse effects and diminish corals’ resilience to human-caused threats. On the other hand, the thresholds we identify are more conservative than the vast majority of species- and region-specific thresholds. In fact, this highlights the utility of our synthetic approach: in the absence of more specific information, we may adopt the most conservative threshold that uses the best available information to protect even the most vulnerable corals from stressful conditions.

*Coral fragmentation* One of the original goals of this systematic review and meta-analysis was to explore how coral morphology contributes to the relative abilities of corals to cope with sediment stress. However, one aspect of experimental design—fragmentation—complicated this kind of synthesis. Experiments with corals, whether in the field or lab, often use fragments or nubbins as their experimental units so that samples are well replicated and reasonably uniform in size/shape. While fragmentation is necessary in most experimental frameworks, one consequence is that fragments often have different shapes or gross morphologies than the parent colony from which they were taken. This is especially true for massive and plating corals, the adults of which have gently sloping or flattened surfaces, respectively, which catch and entrain sediment rain. Coral fragments of massive/plating species are much smaller than their parent colony, such that sediment rain may be more easily removed either through water flow or mucus sloughing. These kinds of differences between coral fragments and whole colonies prevented us from gaining a more mechanistic understanding of how sediment affects corals of differing morphologies. Future studies interested in this question should account for different sizes and growth forms of corals, both within and across species.

*Disentangling co-stressors* Deposited and suspended sediment stressors almost always co-occur but are hypothesized to affect corals by different biological mechanisms. Unfortunately, however, it is logistically difficult to isolate the effects of these two stressors, even in the lab. In fact, no study included in our meta-analyses tested the effects of these stressors both separately and together, and only one experimental study measured total suspended solids (mg/L), turbidity (NTU), light attenuation (relative %), and deposition rate (mg/cm^2^/day) during the course of their experiment [48]. Despite the difficulty of separating these stressors in practice, we separated them analytically based on the unit of measurement that was reported in the text: mg/cm^2^/day was indicative of deposited sediment only, while mg/L was indicative of suspended sediment only. We encourage that future studies be designed to disentangle the effects of deposited and suspended sediment acting separately and in concert.

#### Non-uniformity of study reporting

*Complex coral responses* Our systematic review and meta-analyses describe many different responses of corals to sediment exposure across their life-history and inclusive of both physiological and lethal changes. However, many more articles exist that describe the effect of sediment on coral responses that were inadequately replicated or reported across studies. For instance, bleaching of coral tissue was a common response, but there was little uniformity in how it was reported. Proxies for bleaching included the density of zooxanthellae, the density of chlorophyll-a, the proportion of tissue without zooxanthellae, and indices of tissue paling that were specific to certain regions/species. When possible, we recorded the presence/absence of any bleached tissue as a binary response to be considered in our assignment of NOAELs and LOAELs. Due to the non-uniformities in reporting, however, we could not standardize the differing bleaching responses to investigate the relationship between sediment exposure and the magnitude of bleaching. This lack of comparability in measuring bleaching response was also noted in a recent synthesis and critique of studies focused on coral bleaching [88, 89].

Other less commonly reported responses, like gene expression, were found in too few studies to synthesize, especially considering the ongoing methodological developments in the field. When possible, scientists interested in the effect of sediment on complex coral responses (like bleaching or gene expression) should report some kind of standardized metric that is easily repeatable across species and studies [88]. These metrics will depend on cooperation among scientists in the relevant field, but their creation will prove important in our ability to synthesize evidence across regions, taxa, and scientific labs with differing protocols.

*Quantifying sediment* The specifications of sediment exposure are also often reported inconsistently across studies. Most commonly, the concentration of deposited sediment is reported as mg/cm^2^/day in terms of how much sediment was applied within the area where coral replicates were housed. Less than a third of studies attempted to measure how much sediment came in contact with coral tissue following sediment application, as opposed to remaining in suspension or being swept away by ambient water flow. While this kind of ground-truthing can be logistically difficult even in a relatively controlled laboratory setting, its omission from most study designs complicates comparison across studies in unpredictable ways (i.e., some studies may over- or under-estimate deposition rates). Because of this complication, we took reported dosages of deposited sediment at face value, as best estimates of exposure conditions.

In the case of suspended sediment and turbidity, mg/L and NTU were the most common units of measurement, respectively. Unfortunately, most studies only reported one of these units, and there is no linear relationship between mg/L and NTU. This makes it difficult and potentially misleading to convert from one unit to the other. Therefore, our review and meta-analysis use the results of studies that reported mg/L, and we exclude studies that reported only NTU. We did not do a separate meta-analysis of turbidity (NTU) thresholds because it was reported much less frequently. We recommend that future studies report both mg/L and NTU measurements, whenever possible, so that thresholds for suspended sediment and turbidity can be disaggregated.

Many studies tended not to report much detail concerning the sediment they used in their experiments. There is evidence that corals may be more resilient to stress from coarser, calcareous sediment from marine sources (e.g., “sand”) than from finer, terrigenous sediment from land-based sources (e.g., “mud”) [90]. Unfortunately, however, too few articles consistently reported sediment type or comparisons among sediment types, thus limiting our ability to synthesize trends across studies. Therefore, we recommend that all future studies attempt to quantify (with means and error estimates, when appropriate) sediment dosage, composition, grain size, and other geochemical properties.

#### Interpretation of statistical model results

*Sources of heterogeneity* Great effort was taken to include like-studies and account for potential effect modifiers and other reasons for heterogeneity across studies (see Methods). However, ecological meta-analyses can be fraught with often confounding sources of variability that are either too difficult or numerous to include in the meta-analytical model. In our dose–response meta-analyses, the I^2^ statistic was a measure indicating the percentage of variance in a meta-analysis that is attributable to heterogeneity among dose–response comparisons within a study. Heterogeneity is substantial when I^2^ is above 75%, which was true for several (5 of 11) of our models (Table 7). This may indicate that in many cases, the random effects of species and study were important in determining the relationship between sediment exposure and the magnitude of a coral’s response.

A study species is often confounded with geography and morphology. Most studies are confounded with sediment composition and are not strictly repeatable in the sense that other experimental conditions are. Therefore, the effects we report from our dose–response meta-regressions should be considered as starting points from which data from future studies may clarify and refine the relative roles of sediment exposure vs. experimental context (fixed vs. random effects in a model framework) in shaping corals’ response.

*Gap in tested exposure levels* Sometimes the NOAELs and LOAELs identified were different from the thresholds derived from dose–response meta-regressions of continuous data, challenging our interpretation of model results. For instance, when considering the effects of deposited sediment on photosynthetic efficiency (i.e., maximum quantum yield, measured as *F*_*v*_*/F*_*m*_), the physiological response with the most available data, we find that the NOAEL and LOAEL are 25 mg/cm^2^/day (Table 5) while the dose–response threshold is 3.2 mg/cm^2^/day (Fig. 10C). *Why is it that the dose–response threshold would be so much lower than the lowest reported adverse effect in the literature?* In this case, it is likely because the vast majority of studies focus on exposure concentrations greater than 25 mg/cm^2^/day, with adverse effects occurring even at the lower end of tested concentrations. While the dose–response threshold of 3.2 mg/cm^2^/day is relatively low, it is the result of a meta-regression of effect size by concentration that provides strong evidence that the threshold is outside of the normal range of exposure concentrations. This difference highlights a major gap in our understanding, and the specific need for more studies to be done at exposure levels below 25 mg/cm^2^/day.

#### Review methods

*Search strategy and timing* The choice of search terms that included names of coral genera were selected by our U.S. federal partners, and therefore reflect the taxa that are considered threatened or otherwise important in the Pacific region under U.S. jurisdiction. This may have created a tendency toward U.S.-based studies, but we found little evidence to support this bias given that only 13 of the 65 articles (20%) included in the review and meta-analysis were from the U.S. (Tables 3, 4). Furthermore, all the taxa included in the search term can be found in the Pacific outside of U.S. jurisdiction. While we made efforts to reduce bias against potential sources that were non-English language, our English search terms may have precluded certain languages from appearing in our search results, especially those with non-Latin alphabets including several Asian countries where corals and coral-research is done. Despite this, 17 of the 65 studies (26%) were conducted in locations where English is not the predominant language, 12 of which (18%) were in Asian or African countries.

Our systematic searches of the literature were conducted in mid 2019 and we could not update these searches due to unforeseen challenges relating to the COVID-19 pandemic and personnel changes. Therefore, this systematic review is current as of May 2019 and omits any eligible studies that have been published since then.

*Consistency across review team members* While the protocol for our systematic review is thoroughly described and follows well-established standards [31–33], it is impossible to guarantee 100% consistency among multiple members of a review team throughout the screening, validity assessment, and data extraction process. We implemented protocol that promote consistency and report the results of consistency checking in the Methods.

## Review conclusions

To identify critical threshold values for deposited and suspended sediment on coral reefs, we used a rigorous, peer-reviewed protocol [30] to compile a global dataset that spans three oceans, over 140 coral species, decades of research, and 86 field- and lab-based experiments. To date, sediment thresholds have been estimated from in situ data, where sediment co-occurs with other potential stressors. Rogers [18] observed that ‘normal,’ background levels of sediment on coral reefs are on the order of 10 mg/cm^2^/day for deposition rates and 10 mg/L for total suspended sediment concentrations, above which are considered ‘high’ with the potential to adversely affect corals. Other published critical thresholds on coral reefs range from 37 to 300 mg/cm^2^/day for deposited sediment [86, 91, 92] and from 15 to 260 mg/L for suspended sediment [93–100]. Our review found that adverse effects, including mortality, occur at deposited sediment concentrations as low as 1 mg/cm^2^/day and suspended sediment concentrations as low as 3.2 mg/L (Table 8). The lowest-observed adverse-effect levels (LOAELs) for reduced settlement rates of larvae, mortality of juveniles, and bleached or necrotic tissue of adults were all below 10 mg/L or mg/cm^2^/day. The LOAELs for other coral responses, including sublethal physiological rates and colony mortality of adults ranged between 20 and 40 mg/cm^2^/day for deposited sediment and between 10 and 100 mg/L for suspended sediment. While some of these LOAELs are consistent with previously published critical threshold values above 10 mg/cm^2^/day or mg/L, they also reflect the relative paucity of studies that focus on concentrations below these levels.

In addition to sediment concentration, we also report thresholds for exposure duration. Adverse effects in response to deposited sediment occur on the order of hours to days, while those in response to suspended sediment occur on the order of days to weeks. Generally, we found only modest evidence that coral adults are less sensitive to deposited sediment than are immature stages and no evidence of a developmental change in susceptibility to suspended sediment.

**Table 8:** Summary of LOAEL (lowest-observed adverse-effect level) threshold concentrations and durations of coral exposure to suspended and deposited sediment

Sediment type	Coral response^*^	Threshold^†^ concentration		Threshold^†^ duration	
		Subadults	Adults	Subadults	Adults
Suspended sediment	Any adverse effect	10.0 mg/L	3.2 mg/L	1 h	2 h
	Any mortality	30.0 mg/L	3.2 mg/L	2.5 days	14 days
Deposited sediment	Any adverse effect	1.0 mg/cm^2^/day	4.9 mg/cm^2^/day	3 days	12 h
	Any mortality	8.3 mg/cm^2^/day	4.9 mg/cm^2^/day	3 days	22 h

Coral responses are distinguished between “any adverse effect” and “any mortality,” which are defined in the table footnotes. Subadults are coral gametes, larvae, and juveniles. Adults are reproductively mature coral colonies. All corals in this review are scleractineans

*Any adverse effect is defined as any response of a coral individual, colony, or treatment group that may negatively affect a coral’s fitness and/or survival. These adverse effects may include physiological changes (e.g., reduced growth or photosynthetic rates), bleaching, tissue necrosis, and colony mortality. *Any mortality* is inclusive of death of tissue or of the entire coral colony, and thus excludes sublethal coral responses

^†^Threshold is LOAEL, lowest-observed adverse-effect level, based on the presence/absence of coral response

### Implications for policy and management

The critical threshold values that we identify in our meta-analyses are lower than previously estimated thresholds, which may affect policy and management decisions. Specifically, our thresholds support the implementation of more conservative regulations of both deposited and suspended sediment on coral reefs. The threshold concentrations and durations that we provide will likely prove useful for the community of regulators who are interested in understanding relative risk associated with different levels of sediment exposure near reefs. Our dose–response meta-regressions modeled the relationship between exposure and magnitude of coral responses. While some of these regressions point to thresholds that are similar to identified NOAELs and LOAELs, they mostly highlight gaps in our current understanding of the effects of sediment on a diversity of coral responses.

The kinds of information we provide in this review may allow resource managers to better regulate actions that lead to sedimentation in the nearshore environment, especially dredging and runoff. In fact, this review benefited from several publications associated with the Western Australian Marine Science Institution’s Dredging Science Node, a strategic research initiative that enhanced capacity with government and the private sector to predict and manage the environmental impacts of dredging in their region [101]. Having the best available information about tolerance thresholds of sensitive species will inform the development of standard monitoring protocols and best management practices that will avoid, minimize, and mitigate adverse impacts to the environment from dredging. Runoff is a more diffuse source of sediment pollution that can be challenging to manage. Given how low the thresholds levels are that we identify in this review, resource managers may wish to employ land-sea planning measures that reduce soil erosion rates from different land-use categories, including agriculture, development, roads, mines, and forests [102].

Future experimentation has considerable potential to define more location-specific thresholds that allow for the most defensible regulatory decisions at sites that are most susceptible to sediment-producing events, like dredging and coastal runoff. We recommend protocols for future work (see Implications for research, below), which could be implemented in-parallel across a network of researchers and regulators at multiple locations across a region. During the review process, we flagged experiments that crossed exposure to sediment with that of other common co-stressors, including nutrient enrichment, chemical contamination, and freshwater discharge. However, there were too few of these studies to warrant a co-stressor meta-analysis, as done previously for the effects of temperature and irradiance on corals [103]. A separate systematic review and meta-analysis has quantified the independent effects of chemical contaminants on corals. Thus, additional work should be done to quantify the additive and synergistic effects of multiple local stressors on coral reefs.

### Implications for research

Opportunities to improve future experiments by specifically addressing each of the limitations of this systematic review and meta-analysis were discussed above, in the “13” section. Based on apparent gaps in our understanding and approach-to-date, we make four key recommendations for those interested in defining critical threshold values for sediment on coral reefs:

1. Pair experiments in the lab with those in nearby coastal watersheds to validate estimated thresholds in relevant environmental contexts (i.e., location, species, sediment type, etc.);
2. Target a range of experimental concentrations, between 0.5 and 50 mg/cm^2^/day or mg/L, which should induce physiological and lethal effects in susceptible coral taxa;
3. Standardize reporting of coral responses and stressor dosage/properties, always providing both deposited and suspended sediment levels, including turbidity; and
4. Test for potential synergisms between and among stressors that often co-occur, including deposited and suspended sediment, as well as nutrients, contamination, low salinity, etc.

*Lab experiment* We recommend the use of manipulative experiments to address synergistic effects between deposited and suspended sediment/turbidity, and where possible, among other common co-stressors including light attenuation, nutrient-enrichment, contamination, decreased salinity, and increased temperature or dissolved CO_2_.

Sediment type, exposure levels, taxa, and coral responses that are most relevant at a particular site will be informed by field monitoring and/or associated field experiments. While the set of species chosen for a location may vary, we recommend using populations that are most vulnerable to future disturbances, especially coral colonies found adjacent to the impact zone of a particular stressor. To help account for differences within and among species and geographic locations, we suggest using at least one common species found at multiple locations across a region. Given the general interest in defining sediment exposure thresholds, we also recommend sediment levels between 0.5 and 50 mg/cm^2^/day or mg/L.

To disentangle the effects of deposited and suspended sediment/turbidity, researchers may build a mesocosm array in which coral colonies will be placed in aquaria exposed to different combinations of the two stressors. Stressor interactions will be assessed by monitoring corals under four treatments with all other conditions held at ambient levels: no stressor (control), deposited sediment only, suspended sediment only, and both stressors. In all treatments, deposited sediment and total suspended solids would be measured intermittently, while turbidity and light levels would be measured in real-time.

This kind of experimental setup could be leveraged to quantify multiple responses of control and sediment-exposed corals over the typical duration of a dredging event. Based on the results of our systematic review, candidate responses that could be measured daily are photosynthetic efficiency (*F*_*v*_*/F*_*m*_ with PAM), presence of mucus production and sloughing, and estimates of the percent tissue area that is experiencing either tissue paling (on a location- and species-specific scale), total bleaching, or necrosis. Growth in terms of either change in weight or linear extension rates could be measured on a monthly basis. We further recommend the use of juvenile corals to broaden our understanding of the effects of sediment on immature coral stages that have been less well represented in research-to-date.

*Field experiment* A Before-After, Control-Impact (BACI) design [104] could be used to experimentally track the effects of sediment-producing events on nearby corals. As the acronym suggests, environmental conditions (i.e., sediment deposition rate, total suspended solids, turbidity, and light attenuation) and coral health (i.e., sublethal and lethal effects) could be measured before, during, and after a sediment-producing event at a range of locations inside (impacted) and outside (control) the affected area, as done previously for dredging at other tropical Pacific locations [105, 106].

This kind of study requires cooperation among regulators, scientists, and other stakeholders, but the BACI design is arguably the gold-standard for ascribing causative, in situ relationships between an event and a subsequent biological response. It would also provide on-the-ground monitoring of sediment plumes created by dredging or runoff, a quantitative basis for defining and testing remediation efforts, a range of realistic sediment exposure levels, and a list of vulnerable coral species and populations to be targeted in associated lab experiments.

## Supplementary Information

**Additional file 1.** A filled-in ROSES checklist and meta-data form for this manuscript.

**Additional file 2.** A search scoping exercise and list of definitive reviews and benchmark studies.

**Additional file 3.** The data coding and extraction form to be used for the systematic review and meta-analysis.

**Additional file 4.** Contains the keys to the species codes and coral responses reported in Figs. 4, 5, 6, Tables 3, and 4.

## Data Availability

All data generated or analyzed during this study are included in this published article [and its supplementary information files]. Code used for analyses is available at https://github.com/ljtuttle/coral_stressor_thresholds.
