# Supplementary material for: Effects of sediment exposure on corals: a systematic review of experimental studies
Source: Environ Evid. 2022 Feb 7;11:4. doi: 10.1186/s13750-022-00256-0 (PMC8818373; doi:10.1186/s13750-022-00256-0)
Supplement: Supplementary file 1 — Additional file 1. A filled-in ROSES checklist and meta-data form for this manuscript. [file 13750_2022_256_MOESM1_ESM.pdf]

ROSES for Systematic Review Reports. Version 1.0

November 2017

Neal R Haddaway, Biljana Macura, Paul Whaley, and Andrew Pullin

completed for the following:
Effects of sediment exposure on corals: a systematic review of experimental studies
Additional file 1
Lillian J. Tuttle and Megan J. Donahue
published in *Environmental Evidence*
NOAA grant #NA18NMF4540325 to PI Donahue, "Developing stressor thresholds for reef-building coral species in the Pacific Islands Region"

**Section/sub-section**	**Topic**	**Description**	**Further explanation**	**Checklist/ meta-data**	**Author response**	**Comments**
Title	Title	The title must indicate that it is a systematic review, and should indicate if it is an update/amendment: e.g. "...A systematic review update."	The title should normally be the same or very similar to the review question.	Meta-data	Effects of sediment exposure on corals: a systematic review of experimental studies	
Type of review	Type of review	Select one of the following types of review: systematic review, systematic review update, systematic review amendment, systematic review from a systematic map	See CEE Guidance on amendments and updates [1]	Meta-data	systematic review	
Authors' contacts	Authors' contacts	The full names, institutional addresses and email addresses for all authors must be provided.		Checklist	Yes	

Abstract	Structured summary	The abstract of the manuscript must not exceed 500 words and must be structured into separate sections: Background, the context and purpose of the review, including the review question; Methods, how the review was performed and statistical tests used (specifically mention search strategy, inclusion criteria, critical appraisal, data extraction and synthesis); Results, the main findings, including results of search and assessment of evidence base; Conclusions, brief summary and potential implications for policy/management and research.		Checklist	Yes
Background	Background	Describe the rationale for the review in the context of what is already known. Reviews must indicate why this study was necessary and what it aims to contribute to the field.	A theory of change and/or conceptual model should be presented that links the intervention or exposure to the outcome.	Checklist	Yes
Stakeholder engagement	Stakeholder engagement	The actual role of stakeholders throughout the review process (e.g. in the formulation of the question) must be described and explained (using a broad definition of 'stakeholder', including e.g. researchers, funders and other decision-makers; see [2])		Checklist	Yes
Objective of the review	Objective	Describe the primary question and secondary questions (when applicable).	The primary question is the main question of the review. The secondary questions are usually linked to sources of heterogeneity (effect modifiers).	Checklist	Yes

	Definition of the question components	Provide reference to the question key elements, e.g. population(s), intervention(s)/exposure(s), comparator(s), and outcome(s).	For other question types see [3,4]	Meta-data	**Population**: All life stages of all shallow (photic zone, <=80m depth) scleractinian coral genera in all warm-water ocean basins (20°-30°C).<br>**Exposure**: Exposure to concentrations of suspended and/or deposited sediment of marine or terrigenous origin. For manipulative experiments conducted in either the field or laboratory, this was the application of suspended or deposited sediment.<br>**Comparison**: Specimens experimentally exposed to suspended or deposited sediment must be compared to an appropriate experimental control in either the field or laboratory. Lab controls were often corals not exposed to sediment of any kind and field controls were often corals exposed to ambient conditions without the experimental addition of sediment of any kind.<br>**Outcome(s)**: Specific endpoints are all physical, physiological, behavioral, developmental, and ecological responses of corals	(cont'd) …sediment. These may include but are not limited to tissue or colony mortality, bleaching, and changes in rates of growth, photosynthesis, and larval settlement or survival. Outcomes were recorded as binary or continuous data, as reported in the study.
Methods	Protocol	Provide citation, DOI or open-access link to published protocol.	The protocol should be peer-reviewed and publicly available online (open access).	Meta-data	https://doi.org/10.1186/s13750-020-00200-0	
	Deviations from protocol	Describe any ways in which the final methods of the review deviate from those set out in the protocol along with a justification.		Checklist	Yes	

Searches	Search strategy	Detail the search strategy used, including: database names accessed, dates of searching, institutional subscriptions (or date ranges subscribed for each database), search options (e.g. ‘topic words’ or ‘full text’ search facility), efforts to source grey literature, other sources of evidence (e.g. hand searching, calls for evidence/submission of evidence by stakeholders).	Checklist	Yes

Search string	Provide Boolean-style full search string and state the platform for which the string is formatted (e.g. Web of Science format)	Meta-data	Formatted for Web of Science: ((coral AND sediment*) OR (coral AND suspend*) OR (coral AND turbidity) OR (coral AND mud) OR (coral AND terrigenous) OR (coral AND silt*) OR (coral AND plume) OR (coral AND dredg*) OR (coral AND land-based) OR (sediment* AND Acropora) OR (sediment* AND Anacropora) OR (sediment* AND Cantharellus) OR (sediment* AND Dendrogyra) OR (sediment* AND Euphyllia) OR (sediment* AND Isopora) OR (sediment* AND Montastraea) OR (sediment* AND Montipora) OR (sediment* AND Mycetophyllia) OR (sediment* AND Orbicella) OR (sediment* AND Pavona) OR (sediment* AND Porites) OR (sediment* AND Seriatopora) OR (sediment* AND Siderastrea) OR (sediment* AND Tubastraea) OR (sediment* AND Alveopora) OR (sediment* AND Astreopora) OR (sediment* AND Favia) OR (sediment* AND Favites) OR (sediment* AND Goniastrea) OR (sediment* AND Goniopora) OR (sediment* AND Leptastrea) OR (sediment* AND	(cont'd) …(sediment* AND Lobophyllia) OR (sediment* AND Millepora) OR (sediment* AND Platygyra) OR (sediment* AND Pocillopora) OR (sediment* AND Turbinaria))
Languages - bibliographic databases	List languages used in bibliographic database searches	Meta-data	English	
Languages – grey literature	List languages used in organisational website searches and web-based search engines	Meta-data	English	

	Bibliographic databases	Provide the number of bibliographic databases searched		Meta-data	4	(1) Web of Science, (2) JSTOR, (3)Aquatic Sciences and Fisheries Abstracts (ASFA), and (4) Dissertations & Theses Global (PQDT)
	Web-based search engines	Provide the number of web-based search engines searched		Meta-data	1	(1) Google Scholar
	Organisational websites	Provide the number of organisational websites searched		Meta-data	5	(1) James Cook University Library One Search, (2) ReefBase's Proceedings of the International Coral Reef Symposium, (3) Science.gov (US government database), (4) Great Barrier Reef Marine Park Authority (GBRMPA) Elibrary, and (5) Western Australia Marine Science Institute's Dredging Science Node (WAMSI DSN) repository
	Estimating comprehensivenes s of the search	Describe the process by which the comprehensiveness of the search strategy was assessed (i.e. list of benchmark articles)		Checklist	Yes	
	Search update	Describe any update to searches undertaken during the conduct of the review	Compulsory (if update performed). A search update is good practice if original searches were performed more than two years prior to review completion.	Checklist	n/a	
Article screening and study inclusion criteria	Screening strategy	Describe the methodology for screening articles/studies for relevance. Methods for consistency of screening decisions (at title, abstract, and full texts levels) checking must be described.		Checklist	Yes	

	Inclusion criteria	Describe the inclusion criteria used to assess relevance of identified articles/studies. These must be broken down into the question key elements (e.g. relevant subject(s), intervention(s)/exposure(s), comparator(s), outcome(s), study design(s)) and any other restrictions (e.g. date ranges or languages).		Checklist	Yes
Critical appraisal	Critical appraisal strategy	Describe here the method used for critical appraisal of study validity (including assessment of individual studies and the evidence base as a whole). Describe how repeatability of critical appraisal of study validity was tested.		Checklist	Yes
	Critical appraisal used in synthesis	Describe how the information from critical appraisal was used in synthesis.		Checklist	Yes
Data extraction	Meta-data extraction and coding strategy	Describe the method for meta-data extraction and coding for studies, providing lists of variables that will be extracted as meta-data and those that will be coded. Describe how repeatability of meta-data/data extraction and coding was tested.	Optional, a map database can be included within a systematic review	Checklist	Yes
	Data extraction strategy	Describe the method for extraction of qualitative and/or quantitative study findings. Describe how repeatability of data extraction was tested.		Checklist	Yes
	Approaches to missing data	Describe any process for obtaining and confirming missing or unclear information or data from authors.		Checklist	Yes

Potential effect modifiers/ reasons for heterogeneity	Potential effect modifiers/ reasons for heterogeneity	Provide a list of and justification for the effect modifiers/reasons for heterogeneity that will be considered in the review. Also provide details of how the list was compiled (including consultation of external experts).		Checklist	Yes
Data synthesis and presentation	Type of synthesis	State the type of synthesis conducted as part of the systematic review (narrative only, narrative and quantitative, narrative and qualitative, narrative, qualitative and quantitative, narrative and mixed-methods)		Meta-data	Narrative and quantitative
	Narrative synthesis strategy	Describe methods used for narratively synthesising the evidence base in the form of descriptive statistics, tables (including SM database) and figures. Study findings must only be narratively synthesised and vote-counting must be avoided.		Checklist	Yes
	Quantitative synthesis strategy	If data are appropriate for quantitative synthesis, describe methods for calculating effect sizes, methods for handling complex data, statistical methods for combining data from individual studies, and any exploration of heterogeneity and publication bias. If all studies were not selected for synthesis explain criteria for selection (e.g. incomplete or missing information).	Compulsory (if quantitative synthesis performed)	Checklist	Yes

Qualitative synthesis strategy	Describe methods used for synthesising qualitative data and justify your methodological choices. Describe if and how you plan to analyse subgroups/subsets of data. If all studies were not selected for synthesis explain criteria for selection (e.g. incomplete or missing information).	Compulsory (if qualitative synthesis performed)	Checklist	Yes
Other synthesis strategies	Describe any other approaches used for synthesising data or combining qualitative and quantitative syntheses (e.g. mixed methods) and justify your choice of methodology.	Compulsory (if other synthesis performed)	Checklist	Yes
Assessment of risk of publication bias	Describe methods for examining the possible influence of publication bias on the synthesis.	This may be done for quantitative syntheses using diagnostic plots or statistical tests.	Checklist	Yes
Knowledge gap and cluster identification strategy	Describe the methods used to identify and/or prioritise key knowledge gaps (unrepresented or underrepresented subtopics that warrant further primary research) and knowledge clusters (well-represented subtopics that are amenable to full synthesis via systematic review).	Optional	Checklist	Yes
Demonstrating procedural independence	Describe the role of systematic reviewers (who have also authored articles to be considered within the review) in decisions regarding inclusion or critical appraisal of their own work.	Reviewers who have authored articles to be considered within the review should be prevented from unduly influencing inclusion decisions, for example by delegating tasks appropriately.	Checklist	Yes

Results	Description of review process	Describe the review process including the volume of evidence identified from all sources and retained through each stage of the review. Must also display the number of articles/studies included at all stages of the review in a flow diagram, including the number of articles/studies excluded at each stage.		Checklist	Yes		
	Number of search results	Provide the number of search results from bibliographic databases (including updates if conducted) prior to duplicate removal.	This number should not include web-based search engine or organisational website searches: this will help assessment of the efficiency of the primary search string.	Meta-data	15,006		
	Number of search results after duplicate removal	Provide the total number of search results from bibliographic database searches following duplicate removal.	This number should not include web-based search engine or organisational website searches: this will help assessment of the efficiency of the primary search string.	Meta-data	10,221		
	Full text screening excludes	Additional file containing list of and reasons for full text exclusions.		Checklist	Yes		Additional file 3
	Title screening results	Provide the number of articles retained following title screening.	Optional if screening titles and abstracts together	Meta-data	n/a		
	Abstract screening results	Provide the number of articles retained following abstract screening.	Optional if screening titles and abstracts together	Meta-data	n/a		
	Title and abstract screening results	Provide the number of articles retained following title and abstract screening.	Optional if screening titles and abstracts separately	Meta-data	438		
	Retrieval results	Provide the number of articles retrieved at full text.		Meta-data	396		

Unobtainable articles	Additional file containing list of unobtainable articles.		Checklist	Yes	Additional file 3; in 'full text review tab, the 'reason for exclusion' is marked as 'conference abstract only' or 'not retrieved'
Full text screening results	Provide the number of articles retained following full text screening.		Meta-data	65	
Consistency checking: screening	Results of consistency checking at all stages (screening, data extraction, critical appraisal) must be provided. Provide the number of titles, abstracts and full texts screened and checked for consistency by two or more reviewers as a fraction of the total (e.g. Title: 2000/20000; Abstract: 500/5000: Full text: 10/100).		Checklist	Yes	Noted in the Methods sections, 'Screening process', "Study validity assessment', 'Data coding and extraction strategy'
Critical appraisal exclusions	If any studies are excluded due to low validity, provide the number of studies excluded from further synthesis during critical appraisal.	Compulsory for any studies not included in synthesis due to validity. Reviews authors may prefer to perform a sensitivity analysis (repeating analyses to examine the influence of validity) rather than excluding studies from synthesis.	Meta-data	331 articles were excluded at the full-text screening for a number of reasons relating to the PECO standards, duplication of data with other peer-reviewed sources (e.g., dissertations/theses), language, etc. The reasons for their exclusion are listed in Additional file 3, 'full text review', and many would have been appraised as 'low validity' had they passed this stage. After the full-text screening, there were 0 articles that received a 'low validity' appraisal.	

	Narrative synthesis	Describe the body of evidence identified using figures and tables, avoiding vote-counting (tallying of studies based on results; direction or significance). Each must be presented with descriptive information (meta-data) and extracted study findings. Describe the validity of individual studies and the evidence base as a whole.		Checklist	Yes	
	Extracted data	Additional file containing extracted quantitative or qualitative data (study findings) from included studies.		Checklist	Yes	Additional file 3
	Systematic map database	Additional file containing meta-data and coding for included studies.	Optional, a map database can be included within a systematic review	Checklist	n/a	
	Quantitative synthesis	Present results of quantitative synthesis of study findings (e.g. meta-analysis).	Compulsory (if quantitative synthesis performed)	Checklist	Yes	
	Qualitative synthesis	Present results of qualitative analysis of study findings (e.g. summaries of identified themes or categories). Also provide additional file with the identified themes or categories for each study.	Compulsory (if qualitative synthesis performed)	Checklist	n/a	
	Other synthesis	Present results of any other synthesis methods used.	Compulsory (if other synthesis performed)	Checklist	Yes	
	Risk of publication bias	Describe the results of assessments for the possible influence of publication bias on the synthesis.	For quantitative syntheses this may be done using diagnostic plots or statistical tests	Checklist	Yes	Additional file 3
Discussion	Discussion	Discuss the review results and suggest further enquiry or analysis (e.g. potential reasons for heterogeneity in outcome). Authors may draw attention to specific knowledge gaps.		Checklist	Yes	

	Limitations of the review	Discuss possible limitations in the methods used.		Checklist	Yes
	Limitations of the evidence base	Discuss possible limitations in the evidence base.		Checklist	Yes
Conclusions	Implications for policy/ management	Summarise the state of the evidence base and discuss the way in which the identified evidence may inform policy/practice decision making in relation to the review question. Provide any measure of the uncertainty surrounding the outcome.	Reviews must not include practical environmental management recommendations or advocacy.	Checklist	Yes
	Implications for research	Discuss the way in which the identified evidence may inform research including options for increasing the reliability of study design that could improve future research.	In this section some advocacy for future research on the reviewed topic is permissible provided it is clearly justified by the review outcome/critical appraisal of study validity.	Checklist	Yes
Declarations	Competing interests	Describe of any financial or non-financial competing interests that the review authors may have.		Checklist	Yes

References

[1] Bayliss, H.R., Haddaway, N.R., Eales, J., Frampton, G.K. and James, K.L., 2016. Updating and amending systematic reviews and systematic maps in environmental management. Environmental Evidence, 5(1), p.20.

[2] Haddaway, N.R., Kohl, C., da Silva, N.R., Schiemann, J., Spök, A., Stewart, R., Sweet, J.B. and Wilhelm, R., 2017. A framework for stakeholder engagement during systematic reviews and maps in environmental management. *Environmental Evidence*, *6*(1), p.11.

[3] Collaboration for Environmental Evidence. 2018. Guidelines and Standards for Evidence synthesis in Environmental Management. Version 5.0.

[4] Leeds Institute of Health Sciences. https://medhealth.leeds.ac.uk/info/639/information_specialists/1500/search_concept_tools. Accessed 12/11/2017.
